# Nature‐Inspired Superhydrophilic Biosponge as Structural Beneficial Platform for Sweating Analysis Patch

**DOI:** 10.1002/advs.202401947

**Published:** 2024-06-13

**Authors:** Hanlin Ding, Hao Yang, Seiya Tsujimura

**Affiliations:** ^1^ Department of Materials Science Institute of Pure and Applied Sciences University of Tsukuba 1‐1‐1, Tennodai Tsukuba Ibaraki 305‐8573 Japan

**Keywords:** biosponge, insensible sweat, microfluidics, wearable patch

## Abstract

Perspiration plays a pivotal role not only in thermoregulation but also in reflecting the body's internal state and its response to external stimuli. The up‐to‐date skin‐based wearable platforms have facilitated the monitoring and simultaneous analysis of sweat, offering valuable physiological insights. Unlike conventional passive sweating, dynamic normal perspiration, which occurs during various activities and rest periods, necessitates a more reliable method of collection to accurately capture its real‐time fluctuations. An innovative microfluidic patch incorporating a hierarchical superhydrophilic biosponge, poise to significantly improve the efficiency capture of dynamic sweat is introduced. The seamlessly integrated biosponge microchannel showcases exceptional absorption capabilities, efficiently capturing non‐sensitive sweat exuding from the skin surface, mitigating sample loss and minimizing sweat volatilization. Furthermore, the incorporation of sweat‐rate sensors alongside a suite of functional electrochemical sensors endows the patch of uninterrupted monitoring and analysis of dynamic sweat during various activities, stress events, high‐energy intake, and other scenarios.

## Graphical abstract

An innovative microfluidic patch that incorporates a hierarchical superhydrophilic biosponge enabling efficient capture of various sweat types, including both sensitive and insensitive varieties. This method not only addresses issues of sample leakage but also minimizes sweat volatilization, establishing a reliable approach for the accurate and real‐time collection of dynamic sweat.

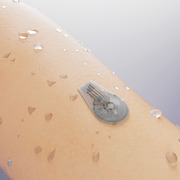

## 1 Introduction

Cutting‐edge, non‐invasive real‐time monitoring platforms, interfacing seamlessly with the skin, are rapidly emerging, among them are microfluidic systems, lab‐on‐a‐chip technologies, and color‐responsive chemistries, which offer tremendous potential for predictive clinical diagnosis. This is critical in combatting a range of prevalent chronic health maladies.^[^ ^1^, ^2^, ^3^ ^]^ The advent of the internet of medical things (IoMT) represents a significant advancement, extending beyond basic tracking to provide essential data on physical movement, biophysical parameters (such as pulse and respiration rates), and metabolite concentrations (including glucose and amino acids). This development encompasses the evolution of next‐generation platforms characterized by flexibility and softness, enabling robust, minimally irritating, and long‐lasting interfaces with advanced materials directly interacting with the human epidermis.^[^ ^4^, ^5^, ^6^ ^]^ Compared to invasive biofluid access, such as blood tests, epidermal perspiration, emanating from eccrine sweat glands, offers promise as a noninvasive, continuous evaluation tool for dynamic physiological status. It furnishes a foundation for evidence‐based health screening, sub‐health assessment, and early diagnosis.^[^ ^7^ ^]^ Significant advancements have been made in addressing critical technical requisites for bioelectronics. These include the development of innovative, ultra‐thin, flexible substrates boasting biocompatibility and reliable, long‐term interfaces. Moreover, the creation of robust biosensors, capable of detecting target analytes at physiologically relevant concentrations within complex samples, has been achieved. These biosensors seamlessly integrate with intricate microfluidic networks, featuring micro‐channels, micro‐reservoirs, and micro‐smart‐valves for precise sample manipulation.^[^ ^8^, ^9^, ^10^ ^]^ Nevertheless, certain emerging wearable platforms, designed for sweat harvesting, directing, and readout monitoring, are tailored for specialized situations, such as minority or strenuous exercise conditions, particularly in athletic, military, or clinical care settings. Their applicability to routine daily life applications is limited due to the requisite specialized digital equipment and highly trained personnel.^[^ ^11^, ^12^ ^]^

In contrast to blood and interstitial fluid, eccrine sweat glands are ubiquitously dispersed across the skin, spanning from the cranial vertex to the plantar sole. Their omnipresence, notably in the form of insensible sweat, manifests as a continuous secretion, serving as a compelling impetus for the exploration of insensible sweat as a conduit for noninvasive health surveillance.^[^ ^13^ ^]^ This involves the employment of small‐scale fiber paper, porous aerogel matrices, or microfluidic patches as agents of noninvasive sweat absorption. The rationale underpinning this approach derives not only from the hypotonic nature of insensible sweat, dominated by water content, but also from its innate profusion of electrolytes, metabolites, exogenous substances, and heavy metal toxins. These biochemical constituents proffer insights into the perspiration rate, body composition, and environmental exposures, encompassing dehydration, electrolyte imbalances, and toxic metal exposures, respectively.^[^ ^14^, ^15^ ^]^ Unfortunately, the customary perspiration emanating from eccrine glands during near‐ or at‐rest thermoregulation occurs at an exceedingly sluggish secretion velocity, oftentimes approximating a diminutive volume of less than 1–5 nanoliters per minute per gland. This scenario poses formidable challenges for continuous monitoring, exacerbated by the attendant phenomena of evaporation and potential contamination.^[^ ^16^ ^]^ In contrast, heightened physical exertion, elevated ambient temperatures, or strenuous activities instigate copious perspiration, thereby affording wearable patches a short window to harvest ample sweat samples expeditiously. Notably, our bodily perspiration reflects various physiological states, including thermoregulation, sedentary behavior, routine or irregular metabolic fluctuations, emotional responses, and pathological manifestations. Traditional methods necessitate a high passive sweat venting rate, typically induced by rigorous physical training or external stimuli. Consequently, translating data gleaned from these aforementioned sweat samples into practical applications for subjective self‐assessment or real‐world health evaluation beyond the confines of a controlled laboratory environment becomes a formidable endeavor.^[^ ^17^, ^18^ ^]^ Nonetheless, the realm of continuous insensible sweat monitoring and analysis remains an enticing and pivotal frontier, albeit one fraught with formidable challenges.

Nature stands as a boundless reservoir of inspiration, teeming with ingenious exemplars that have been perfected through aeons of evolution and optimization. A preeminent example lies in the intricate mechanisms governing the conveyance of fluids and vital nutrients within plant vascular networks. This natural marvel orchestrates multiphase transport and intricate reaction processes, facilitated by meticulously engineered porous and hierarchical structures. Remarkably, humanity has harnessed and re‐engineered these botanical blueprints, repurposing them for an array of transformative applications, including separation technologies, adsorption phenomena, mechanical reinforcement, and tissue engineering endeavors.^[^ ^19^, ^20^ ^]^ These natural mesoporous structures, spanning from random to ordered hierarchies, bestow upon them exceptional attributes in terms of fluid dynamics, transport efficiency, and material collection capabilities endowing materials with distinctive anisotropic mechanical properties.^[^ ^21^ ^]^ To date, sophisticated hierarchical architectures, extending from the nano‐ to the micro‐structure, have found wide‐ranging utility in modulating surface wettability. This modulation effectively governs the spontaneous and directional movement of fluids, dictated by the asymmetric interplay of surface tensions.^[^ ^22^, ^23^ ^]^ A departure from superhydrophobic and engineered gradient surfaces,^[^ ^24^, ^25^ ^]^ these ordered porous frameworks exhibit superhydrophilic attributes, enabling rapid liquid absorption and dispersion in mere microseconds.^[^ ^19^, ^26^ ^]^ Hence, the formulation of a well‐designed continuous and spontaneous liquid transport system represents a promising avenue to bridge the gap in the uninterrupted collection of insensible sweat.

Here, we employed an interconnected hierarchical biopolymer‐based sponge featuring a micro‐nano‐scale fibrillar structure to augment a wearable patch for rapid sampling of natural sweat, especially for insensitive types, and eventually in achieving the continuous monitoring of target biomarkers and easy measurement of secretion rates. While flexible porous substrates have been widely employed to enhance the fabrication of high‐performance electronics, such as e‐skins, nanogenerators, and implantable medical substrates.^[^ ^16^ ^]^ This is the first time that flexible porous superhydrophilic matrices have been synthesized into sponge channels and seamlessly integrated within a microfluidics system. This approach not only drastically reduces the requisite collection time and inlet hydraulic pressure for sweating

## 2 Results and Discussion

### 2.1 Design of Epidermal Wearable Patch

**Figure 1:**
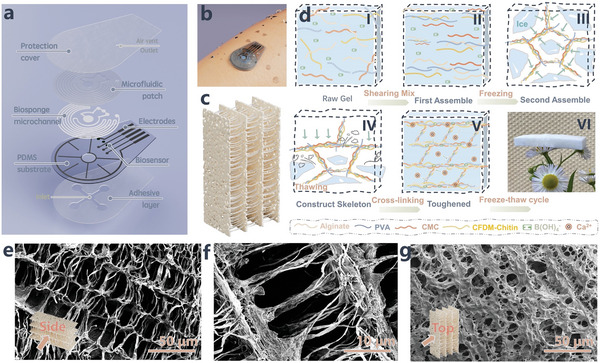
Schematics of wearable biosponge microfluidic system, biosponge synthesis process and its internal morphology. a) Exploded‐view illustration unveils tailored microfluidic system featuring laminated biosponge microchannel, boosting capabilities of sweat monitoring and in‐situ analysis. Superhydrophilic biosponge swiftly seizes upon freshly secreted natural perspiration, channeling it onto purpose‐built biosensors for seamless continuum of real‐time analysis. b) Conceptual illustration portrays fully assembled patch securely affixed to skin's surface. c) A sketch of biosponge channel segment, featuring its biomimetic hierarchical skeleton geometry. d) Biosponge is constructed via two‐step lyophilization assembly process. Initially, a homogeneous raw gel (I to II) is created which undergoes the first lyophilization stage to form an initial rigid and robust skeleton through interpolymer hydrogen bonding (III to VI). Subsequently, a crosslinking and salting‐out process (V) is employed to foster additional interactions between ions and polymer chains, which augment the stiffness and toughness of the skeletal framework. The following implemented lyophilization treatment ultimately results in the establishment of a final biomimetic uniformly distributed hierarchical framework (V). Scanning electronic microscope (SEM) images reveal distinct perspectives of biosponge: e) side view, f) evenly distributed supporting construction, and g) top view.

The disassembly diagram in **Figure** **1**a,b illustrates the composition of the flexible epidermal wearable patch, designed to facilitate continuous monitoring and access to insensible sweat. This device comprises a multilayer stack, encompassing three distinct subsystems, each meticulously engineered to fulfill its designated role:

Low‐modulus biocompatible polydimethylsiloxane (PDMS) with designed geometrical features defining the upper microfluidics layer and bearing the down electrode layer. The standard soft lithography method is manipulated to generate the geometrical features of the mosquito coil shape on the microfluidics layer which allows for the efficient harvest ≈2000 nanoliters of sweat. This quantity proves more than sufficient for continuous monitoring spanning over 24 h, encompassing dynamic perspiration corresponding to routine activities (typical sweat rate range: <1–20 nanoliters per minute per gland).^[^ ^27^ ^]^ The microfluidics layer further incorporates a specific circular region, measuring 2.5 mm in diameter and ≈100 µm in thickness, which serves as the inlet, establishing direct contact with the eccrine glands on the skin.

The electrode circuitry is seamlessly bound onto the bottom PDMS layer and various types of circuits for integrating functionalized electrochemical sensors including glucose, uric acid (UA), [Na^+^] and pH, [K^+^] and pH measurement that can be manipulated using transfer print or screen‐printing techniques.^[^ ^28^ ^]^ The comprehensive circuitry, activated by sweat, is modulated to facilitate sweat rate prediction and is culminated with an outer unenclosed circular region and a central interdigitated wheel circuit component.

The versatile biosponge layer focused on rapidly absorbing varying quantities of sweat is the pivotal component in our study. The final compact wearable patch is sealed with a medical‐grade bandage, ensuring secure attachment to any area of the subject's body, from high sweat secretion regions to those with minimal activity. This feature enables continuous monitoring without disrupting subject's daily activities, rendering it an ideal platform for routine users to gain insights into their physiological status.

### 2.2 Design of Epidermal Wearable Patch

Interfaces possessing hydrophilic characteristics, coupled with porous skeletal structures, exhibit a remarkable predisposition for facilitating spontaneous and directional liquid transport.^[^ ^22^ ^]^ Inspired by the natural intricate geometric shape of the trunk's xylem,^[^ ^29^ ^]^ we devised a superhydrophilic hierarchical biosponge, as depicted in Figure 1c, characterized by an exceptionally robust affinity for water. This innovative design seeks to surmount the challenges associated with capturing and retaining sweat, including issues such as time‐consuming processes, contamination risks, high flow velocities, demanding volume requirements, and inadequate hydraulic pressure loss.^[^ ^27^, ^30^, ^31^ ^]^ The conventional ice‐templating method is widely used to emulate isotropic porous and hierarchical structures. However, the supramolecular biopolymer generated through this method, entailing lyophilization and spontaneous polymerization, is susceptible to significant volume contraction, typically ranging from 25% to 60%. This is due to a lack of stable coordination between the carboxyl group and the divalent ion.^[^ ^32^ ^]^ Furthermore, during the gradual evaporation of ice crystals, portions of the skeleton structure may prove incapable of withstanding their own weight, resulting in internal collapse. To mitigate this, a modified carboxymethyl fiber‐like deacetylation mycelial chitin (CFDM‐Chitin) extracted from fungal cell walls is incorporated to enhance the properties of the pristine biosponge and act as a structural reinforcement composite to fortifying the backbone network. The biomimetic biosponge has been meticulously crafted with aligned isotropic porous and hierarchical skeletons, driven by the following considerations:

- The incorporation of a chemosynthetic porous aerogel, derived from bio‐based materials, forms the fundamental scaffold. This aerogel exhibits an expansive specific surface area, characterized by low density and exceptional porosity, enabling optimal utilization of the channel's volume without causing obstructions and ensuring the rapid water permeation.
- The CFDM‐Chitin binder points consist of a linear polysaccharide composed of β‐(1→4)‐linked D‐glucosamine (deacetylated unit) and N‐acetyl‐D‐glucosamine (acetylated unit). These fibers assume a pivotal role, engaging in covalent cross‐linking through a combination of inter‐ and intramolecular reactions,^[^ ^33^ ^]^ leading to the formation of dual networks and supramolecular assemblies with algal polysaccharides. Consequently, the internal porous structure undergoes reconfigured, enhancing mechanical strength and structural stability. Furthermore, these fibers act as functional components within the meticulously constructed hierarchical supporting structure.
- The distinctive positively‐charged fibers and amino functional groups endowed with antibacterial properties bestow the biomimetic biosponge with remarkable biocompatibility and an inherent resistance to biofouling, thus, reducing the potential bacterial contamination of the long‐term sweat sample retention.

The evolutionary progression of the biomimetic sponge is visually depicted in Figure 1d, achieved through a multistep top‐down‐freeze‐drying method and a sequential ionic‐crosslinking fixation process. The initial pristine sponge (Figure S1a, Supporting Information) acts as the foundational substrate upon which the initial porous morphologies take shape, resembling lamellar structures (as illustrated in Figure S1b, Supporting Information). The eventual transformation into a highly functionalized biosponge, boasting an intricately orchestrated structure, was achieved through the incorporation of additional CFDM‐Chitin, which reinforced the double‐layer backbone of “pristine sponge” while simultaneously acting as a binder to establish pivotal bonding points.^[^ ^32^, ^34^ ^]^ Inset images (Figure 1e–g) reveal morphology of the resultant biosponge configuration, showcasing hundreds of distinctive fiber pillars interspersed within the spaced hierarchical skeletal framework. Additionally, the prepared biosponge exhibited an innate proclivity to rapidly absorb liquids, owing to the high osmotic pressure engendered by its porous structure and the abundance of hydrophilic side groups within its polymer chains. Exploiting the inherent attributes of this skeletal structure and its innate superhydrophilic, any fluid, specifically sweat once secreted and encountered with the biosponge, triggers an ultra‐rapid dispersion, saturating the surface within mere microseconds. This remarkable phenomenon can be ascribed to the biosponge's ultralow contact angle hysteresis, as illustrated in Figure S2 (Supporting Information), and its minimal resistance to liquid flow.^[^ ^16^ ^]^ This xylem‐like architectural configuration, combined with its superhydrophilic interface, serves as a robust reinforcement for accelerating capillary flow, endowing it with adaptive and spontaneously accelerating performance, essential for efficient sweat absorption and transport.

### 2.3 Anti‐Gravity Pumping and Spontaneous Fluid Collection

**Figure 2:**
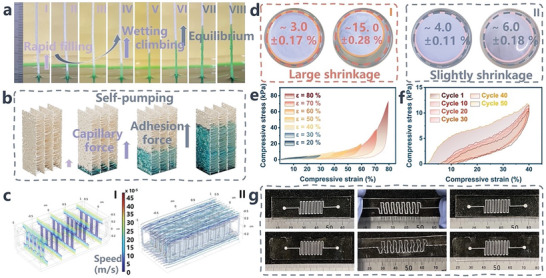
Artificial long‐strip biosponge for fast liquid absorption, predictable theoretical modeling, and superior mechanical performances. a) Optical images of liquid self‐pumping progression. b) Longitudinal cross‐section illustrates phenomenon of self‐pumping in simulations within tubular fluidic structure featuring hierarchical skeleton microchannels. c) COMSOL Multiphysics simulation ascent of fluid within TFS column. Positioning of liquid meniscus is influenced by capillary force, adhesion strength, and gravitational force, all of which determine velocity of liquid advancement and speed distribution on internal (I) and surface (II). See Movie S1 (Supporting Information). d) Comparison of approximate shrinkage ratios between Sponge I and Biosponge after undergoing two cycles of freeze‐drying in identically sized culture dishes, respectively. e) Stress–strain curves during compressive cycles of biosponge, with strain (ε) ranging from 20% to 80%. f) 50‐cycle fatigue test conducted under 40% compressive strain. g) Demonstration of device with two types of U‐turnshaped biosponge microchannels, showcasing maintenance of their structural integrity even after enduring multiple cycles of stretch‐induced deformation.

The phenomenon of capillary liquid ascent within open architectures, involving the interplay of liquid‐solid and gas‐liquid interfaces, is notably intricate in comparison to fluid transport in simple microtubes, where cohesive forces within the liquid and adhesive forces at the hydrophilic surface interface mainly drive the flow. To investigate the capillary liquid transportation within an open architecture at static equilibrium, we shaped the biosponge into a strip shape. As shown in **Figure** **2**a, when the lower part of the superhydrophilic surface contact with the liquid level, a combination of capillary pressure and liquid adhesion prompt the liquid to infiltrate the superhydrophilic architecture, spreading throughout it. This engenders an upward liquid climbing, creating an “anti‐gravity” self‐pumping phenomenon. This process allows for the liquid to ascend a considerable distance against gravity (Figure 2a VIII). The capillary rise of the liquid within this open architecture unfolds in distinct phases: rapid filling (Figure 2a I–IV), wetting climbing (Figure 2a IV–VI), and eventual equilibrium (Figure 2a VI–VIII). During the initial phase, the surface tension of the liquid results in a significant upward capillary force, overpowering other contributing factors and thereby dominating the ascent. As the liquid reaches a certain height, the influence of gravity gradually becomes more pronounced, leading to a deceleration in the liquid pumping process. At this point, adhesive forces along the vertical walls play a pivotal role, promoting the final stages of liquid pumping due to the inherent wettability of the superhydrophilic interface, eventually reaching a pseudo‐equilibrium phase. We used COMSOL Multiphysics simulations, as illustrated in Figure S3 (Supporting Information), to depict the self‐pumping effect occurring when a porous medium contacts a fluid. These simulations confirm that absorption driven by capillary forces continues until an equilibrium is achieved. At this equilibrium, gravitational forces offset capillary forces, aligning with the behavior observed in our long strip biosponge regarding liquid absorption.

To extend this concept effectively to microfluidic patches, further exploration of the steady‐state analytical mechanism of capillary flow in open architectures with superhydrophilic channels is required. We can model the biosponge strip as a column, specifically a stacked column with dimensioned tubular fluidic structures which serves as a critical component, referred to as a TFS column (as illustrated in Figure 2b; Figure S4, Supporting Information). Combined with the simulation results (Figure 2c), to validate the theoretical models align with practical observed phenomena and provide a more applicable macroscopic description of capillary flow and fluid behavior within the microfluidic system, which in turn offers valuable insights for the development and optimization of the microfluidic patch. In a superhydrophilic TFS column, a meniscus forms between two vertical walls, providing the primary capillary force that appears immediately and generates a capillary flow. This flow is supported by an adhesion force provided by the superhydrophilic interface, enabling the liquid to climb upward against gravity until reaching a certain height where the forces are in equilibrium. A longitudinal section view (Figure 2b,c) is used to analyze the upward liquid motion against gravity. The variation in climbing height correlates with the column's specifications. While enlarging the cross‐sectional area results in increased infiltration area and more adhesion force, a thicker column introduces more liquid volume, which creates additional gravitational resistance. However, the prominent meniscuses on each side provide additional asymmetric Laplace pressure (*ΔP* = *γ/R*), and the wetting of the increased surface area offers more adhesion force. This factor is crucial in determining the equilibrium point, leading to higher capillary rise. It's noteworthy that even an extremely fine superhydrophilic sponge possesses the capability to facilitate spontaneous liquid absorption and transport against gravity over considerable distances (as shown in Figure S4, Supporting Information), making it ideal for passively guiding microscale volumes of sweat and pumping it into the microchannel system. However, transpirational pull, driven by non‐steady‐state conditions in transpiration, represents another critical factor in liquid transport. In microfluidic systems, the gas‐liquid interface assumes a complex position and is highly dependent on temperature gradients, especially within the air‐exposed open structure. This complex interplay of factors extends over long timescales.^[^ ^19^, ^35^ ^]^ Considering the potential impact of evaporation, which can compromise the secure storage of sweat within a microchannel, designing a sealed pathway with an array is necessary for effective and accurate sweat analysis.

Transforming raw gel materials into aerogels typically involves significant volume shrinkage after a series of steps including freezing, thawing, solvent exchange, and ambient drying.^[^ ^32^, ^36^ ^]^ To address these challenges and realize our concept, we synthesized a biomimetic biosponge inspired by xylem structures. This biosponge boasts a stronger backbone with a hierarchical structure, along with improved physical and mechanical properties. It is designed to maintain its integrity during the lyophilization cycle and play a pivotal role as a superhydrophilic and anti‐bacterial element for efficiently drawing sweat into the harvest chamber, a key advantage in our study. Figure 2d compares the contraction during the lyophilization cycle is presented for two groups of biosponges. Sponge I comprises alginate (SA) and polyvinyl alcohol (PVA), forming the initial layer of a double‐network structure that contributes to a robust backbone that exhibits excellent shape retention with only a 3.12% contraction ratio (Figure 2dI). The presence of PVA chains plays a crucial role in hydrogen bonding interactions, which slow down the rate of ice crystal growth and promote the formation of micro‐ice crystals, ultimately leading to the creation of a specific aligned isotropic porous structure. After the ice crystals are formed, boric acid (BA), with a relatively low pKa value, donates protons to the hydrophilic (‐OH) side‐groups on PVA, forming borate ester linkages and giving rise to a 3D hierarchical network.^[^ ^37^ ^]^ The resulting hierarchical skeleton boasts a highly porous structure, which is further stabilized by the sublimation of ice crystals during the lyophilization process and is further reinforced by soaking in a salt solution (4.0 wt.% CaSO_4_). However, the pre‐existing polymer chains within this composition contain carboxylate groups. These groups engage in interactions with Ca^2+^ ions through ionic cross‐linking, resulting in a more compact aggregation of the polymer chains and a reduction in pore size within the structure While this secondary assembly significantly enhances the mechanical properties of the biosponge, it also results in an overall reduction in the sponge's volume, measuring ≈15.62% after another lyophilization process, as illustrated in Figure 2d II. In contrast, CFDM‐Chitin and carboxymethylcellulose (CMC), both of which contain abundant carboxyl groups, play a crucial role as reinforcement materials. They form stable coordination through a freeze‐induced physicochemical cross‐linking process, further enhancing the mechanical properties of the biosponge. They also intertwine and intercalate with algal polysaccharide networks, allowing for supramolecular assemblies with multivalent ions to lock SA and PVA networks, rendering them even more robust and stable. Consequently, the biosponge experiences a minimal shrinkage of less than 4.06% (first assemble) and 6.01% (second assemble), respectively, as demonstrated in Figure 2d.

The cuboid‐shaped biosponge exhibited a low density of 14.5 mg cm^−3^. To further characterize its mechanical properties, a unidirectional compression test was conducted, and the results were compared with those of pristine Sponge I. In the case of biosponges, a typical hierarchical monolith reveals three characteristic regions on the stress‐strain curves during uniaxial plane compression (Figure 2e). The cyclic compression test showed plastic deformation, evidenced by the hysteresis curves (Figure 2f), this can be attributed to the collapse of less stable or unabridged fiber‐pillars within the laminated skeleton. Notably, the first compression cycle exhibits a relatively higher energy loss coefficient of 43.6%, although it still retains ≈90% of the initial stress (Figure S5a, Supporting Information). In contrast, the biosponge demonstrated excellent durability in cyclic performance, with no significant reduction in strength observed even after the second compression, extending up to over 50 cycles. Likewise, Sponge I exhibits favorable mechanical stability without structural collapse during the experiment. However, when subjected to external forces, it eventually experiences structural failure with fracture breakage and yields a tensile strength of 124.43 kPa. This value is approximately two times lower than that of the hierarchical structurally enhanced biosponge, which demonstrates an impressive tensile strength of up to 198.32 kPa (Figure S5b, Supporting Information). To further confirm the structurally reinforced networks and evaluate the ability to withstand deformation while preserving the skeletal integrity, the biosponge was placed within microchannels (Figure S6, Supporting Information). As anticipated, the pristine Sponge I, composed solely of SA/PVA, experienced substantial volume shrinkage and struggled to maintain its network structure. In contrast, the biosponge, formulated with the optimized recipe, demonstrated exceptional fatigue resistance and improved mechanical stability, effectively counteracting this shrinkage. This underscores the material's ability to strike a balance between mechanical strength and tensile resistance, maintaining its structural integrity even under conditions of shrinkage.

The synthesized biosponge is integrated within microchannels to investigate the structurally reinforced networks and determine whether the deformation can be accommodated while maintaining the skeletal integrity. Figure 2g and Movie S2 (Supporting Information) illustrate the biosponge in two different microchannels that return to their initial state without experiencing any fracture or shape failure after the applied tensile was released. In contrast, the control group of Sponge I in Figure S7 (Supporting Information) exhibits minor defects, which serve as weak points compromising its ability to withstand stretching forces. Moreover, the superior speed at which liquid is harvested under identical injection speeds provides clear evidence that the superhydrophilic interface skeleton within the microchannel efficiently transports fluids and enhances sampling efficiency (Figure S8 and Movie S3, Supporting Information), which is highly attractive and offers an additional opportunity for effectively capturing small volumes of body fluids.

### 2.4 Thermoregulatory Sweat Collection of Microfluidic Patch

**Figure 3:**
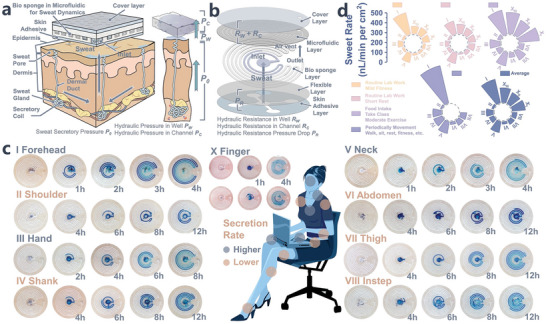
Prolonged monitoring of routine sweat rates through biosponge microfluidic devices positioned across various body regions. a) Anatomical depiction delineating eccrine sweat gland and b) coupled with schematic mirroring a biosponge microfluidic device designed for purpose of precise and efficient sweat harvesting. c) Human trial of sweat‐rate monitoring, blue‐dyed biosponge within microchannel serves to delineate sweat harvesting across diverse body locations over time intervals, depicted in optical images. Employment of variant patch dimensions is tailored to ensure optimal fit for distinct monitoring regions, complemented by using an inlet zone with diameters ranging from 2.5 to 5 mm. d) Circular bar graph signifies computed average sweat rates across represented regions through optical monitoring of sweat volume within microchannel, derived from scrutinized images captured in c).

Leveraging insensible sweat as a non‐invasive medium for biomarker access holds promise but presents a significant challenge. In contrast to the more readily observable sweat (sensible) triggered by physical, thermal, and emotional stimuli, insensible sweat is secreted at an exceedingly low rate, primarily as vapor, which quickly evaporates. This rapid evaporation hampers the collection of sufficient biofluid volumes for analysis by wearable devices. The eccrine gland, illustrated in **Figure** **3**a, can function as a capillary pump integrated into the existing microfluidic system, directing liquid onto the skin beneath our patch's covering. For instance, gland secretion pressure, when chemically stimulated, can reach an impressive 72 kN/m^−2^ in‐vivo, nearly doubling the secretion rate (20–100 nL min^−1^ per gland) compared to normal conditions (5–10 nL min^−1^ per gland).^[^ ^27^, ^38^ ^]^ Designing the microchannel dimensions to efficiently and continuously measure sweat becomes essential. Striking a delicate balance is necessary to minimize excessive fluid resistance while ensuring adequate capacity, thus facilitating uninterrupted perspiration collection from various sweat glands operating under varying secretion pressures. However, as per Poiseuille's flow theory, in specific geometries, longer flow paths entail greater fluid resistance, potentially obstructing the continuous entry of sweat into the microchannel pathways.^[^ ^16^, ^27^ ^]^ To overcome these challenges and achieve swift, uninterrupted sweat collection within constrained dimensions, a superhydrophilic biosponge was introduced into the microchannel (Figure 3b). This innovative approach effectively addresses challenges related to hydraulic resistance and losses, enhancing the system's overall efficiency.

The sandwich‐structured architectural design depicted in Figure 3b features a biosponge embedded within the fluidic conduit, demonstrating exceptional wetting properties. This biosponge swiftly absorbs in‐vitro perspiration, including its vapor phase, ensuring its smooth entry into the channels without encountering excessive resistance. Simultaneously, it also prevents leakage by mitigating excessive hydraulic resistance at the inlet, facilitating seamless conveyance within the microchannels. Furthermore, the hermetic configuration and uniform distribution effectively prevent the volatilization of harvested sweat, ensuring efficient and reliable sweat collection. To accommodate the varying resting sweat secretion rates across different body areas, we conceived two patch variants, adept at amassing and detecting more than 2000 nanoliters of sweat. The detailed specifications of the patch structure can be found in the Supplementary Information (device design layout, Figure S9 and uniformity of biosponge, Figure S10, Supporting Information). The fluidic architecture, reminiscent of a mosquito coil, has been intricately engineered to optimize ample sweat collection within confined spaces. This design not only facilitates thorough sweat harvesting without impeding sweat gland secretion but also assumes a pivotal role in precisely directing the sweat onto the interdigitated electrode array. This enables selective and continuous quantification of sweat rates across the expansive sensor footprint. Thus, this ingenious design ensures enhanced adaptability and precise monitoring of sweat secretion rates. To further authenticate the efficacy of our microfluidic patch, we conducted experiments across various body regions to monitor perspiration rates and volumes, as elucidated in Figure 3c,d. These regions encompassed the forehead, neck, shoulders, arms, abdomen, and lower extremities. We also designed more diminutive patches to accommodate areas exhibiting elevated perspiration rates but constricted dimensions, such as the interdigital spaces of the fingers (Figure S9, Supporting Information, dimension of variant). Subjects were instructed to minimize vigorous physical activities and capture optical images for real‐time sweat collection at various intervals. The use of a blue dye facilitated better observation of the sweat‐advancing footprint. It's worth noting that while a larger inlet coverage area can accommodate more sweat glands, it concurrently creates a lagging region (stagnant pit) that necessitates the filling of perspiration before ingress into the monitoring zone. The time to occupy this region varies based on the sweat rate. For instance, higher sweat rates on the forehead, fingers, and chest could saturate the region within ≈1 h, whereas other regions manifesting lower perspiration rates may necessitate more protracted intervals to reach saturation. To facilitate comprehensive analysis, we partitioned the circular collection expanse into eight equally sized sectors (Figure S11, Supporting Information), thereby enabling the assessment of corresponding perspiration rates and progressions. The average secretion rates at different locations (as shown in Figure 3c) are documented in Table S1 (Supporting Information), with detailed calculation methods provided in the supplementary information. The computed results provide evidence that sweating rates on the brow and fingertips (ranging from 1–20 to 1–30 µL min^−1^ per cm^2^) markedly surpass those discerned at alternative collection sites (1–10 µL min^−1^ per cm^2^), aligns with literature indicating higher density of eccrine glands concentrated around the head, upper body, and palms.^[^ ^39^, ^40^ ^]^ Our biosponge‐equipped patches effectively collected and monitored sweat even in regions with diminished secretion rates and fewer glands, such as the forearm, abdomen and legs. Over a duration spanning more than half a day of monitoring, these patches exhibited no discernible signs of leakage. In contrast, the pure channel patch experienced extensive leakage due to inadequate sweat gland secretion pressure and hydraulic losses, as depicted in Figure S12 (Supporting Information).

Quantifying the sweat loss rate is crucial for detecting dehydration in individuals. However, the accurate evaluation of overall bodily perspiration rates and, by extension, the severity of dehydration based solely on data from wearable patches remains a significant challenge. To tackle this, we conducted a trial concentrating on the brow, chest, and upper limbs as monitoring sites, representing regions with high, medium, and low sweating rates, respectively. Subjects were instructed to initiate moderate‐to‐vigorous physical activity to amass sweat volumes from these specified regions, which were then compared to the overall body sweat loss. In general, the manifestation of mild dehydration symptoms becomes evident when the loss of bodily weight attributable to dehydration exceeds 1% to 2%.^[^ ^41^ ^]^ Following engagement in medium‐intensity physical exertion without fluid intake for a duration surpassing 30 min, subject reported conspicuous sensations of weariness and thirst. Concurrently, the cumulative body perspiration loss ≈500–700 ml (in 60 min), coinciding with the monitoring collection circles delineated by our wearable patch at positions 3 (upper limbs), 5 (chest), and 6 (brow) circles (as indicated in Figure S13, Supporting Information). Consequently, we can subdivision the collection region into three monitoring sections: the standard sector, mild dehydration sector, and perilous sector (Figure S13c, Supporting Information), whereby serves as a reminder for individuals, signaling whether they should expeditiously replenish fluids or electrolytes. Further research is necessary to minimize and catalog interindividual physical variances, aiming to establish an indicator for continuous monitoring of sweat that can progressively achieve enhanced accuracy and reliability. This will refine the monitoring process, ensuring more consistent and dependable results.

The strategy of continuously tracking and monitoring routine sweat rate and sweat loss is intriguing, as an alternative approach, our microfluidic system, coupled with biosponge serves as a vehicle for providing direct visual readout, offering an auxiliary “wireless” monitoring avenue (Figure 3c; Figure S13c, Supporting Information). Undoubtedly, there are several factors to consider when it comes to potential interfering factors in sweat monitoring. First and foremost, accumulation of sweat beneath the adhesive layer may cause transudation‐type leakage, thereby resulting in an underestimation assessment of the actual sweat volume collected. Second, sweat glands that are obstructed by the adhesive layer may experience a diminution in secretion rate. However, this can potentially trigger a compensatory mechanism, resulting in accelerated sweat gland secretion in uncovered areas, maintaining overall sweat production.^[^ ^42^ ^]^ Furthermore, the presence of the adhesive patch on the skin may hinder heat transfer, affecting sweat production in the covered region. The thermal imaging depicted in Figure S14 (Supporting Information) demonstrates that our lightweight and compact biosponge patch has a negligible influence on localized skin heating, in contrast to relatively bulky patches that exhibit noticeable temperature differences due to the obstruction of heat conduction. To further validate the accuracy of our sweat monitoring patch, we conducted a comparative analysis against a conventional sweat detection method and quantified sweat volume and rate across three different secretion velocities (employing the brow, chest, and upper limbs as monitoring areas) and normalized the results to the unit area by engaging in moderate‐intensity physical activity. The sweat rate readouts obtained from the biosponge patch were ≈2.4 times higher than those recorded by conventional absorbing pad (Figure S15, Supporting Information).^[^ ^43^ ^]^ Notably, our device allows for the continuous capture of sweat information without the need for patch removal and gravimetric analysis during each experiment, as the secreted sweat is immediately absorbed by the hydrophilic biosponge and guided into the microchannels, enabling visual monitoring and preventing fluctuations in sweat evaporation that can occur in conventional measurement methods. The continuous monitoring of the regional (brow) sweat rate, compared with the systemic sweat loss during prolonged distance jogging, the patch with biosponge exhibited a better correlation (*R* ^2^ > 0.90) to those results obtained with the absorption pad (*R* ^2^ > 0.67) (Figures S15a and S16, Supporting Information). This demonstrates the advantages of our patch, particularly in the realm of continuous monitoring and sweat collection. Furthermore, the extended‐duration continuous monitoring images presented in Figure 3 provide evidence that there is no obvious lateral leakage or displacement of sweat in the collection wells. These results support the hypothesis that the sweat collected by our patch closely reflects the actual localized sweat rate, offering a viable option for continuous sweat monitoring in practical scenarios.

### 2.5 Evaluation for LEG‐Based Sweat‐Rate Sensor and Multi‐Biomarker Sensor Analysis

**Figure 4:**
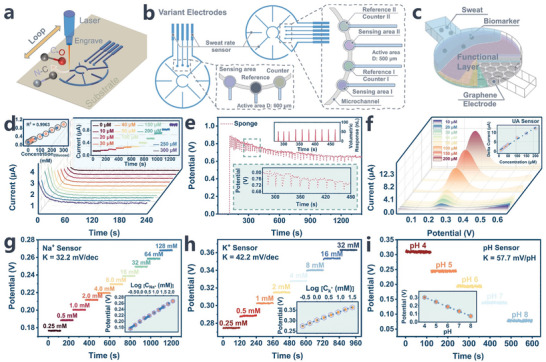
Production methodology of laser‐engraved graphene‐based variant electrode circuit and subsequent assessment of sweat sensor's performance. a) Schematic of laser‐engraved circuit. b) Layout of two representative circuits delineating sweat sensor components. c) Mechanism elucidating analysis of sweat biomarkers. Comprehensive sensor test results and corresponding calibration plot demonstrating efficacy of sensor in measuring d) glucose, e) sweat rate, f) uric acid, g) sodium, h) potassium, and i) pH sensor were conducted using an electrochemical workstation and standard analyte solutions.

Our objective is to enhance the functionality of sensing electrodes metamorphosing them to achieve uninterrupted and efficient monitoring of both sweat rate and specific biomarkers. By introducing a diverse array of electrode configurations, in synergistic pairings with a microfluidic biosponge system, we endow our microfluidic patch with the versatility to seamlessly accommodate a wide spectrum of individualized health monitoring requisites. Graphene, known for its distinctive electrochemical properties, has been utilized as a substrate for high‐performance biosensors capable of detecting electroactive analytes at extremely low levels.^[^ ^44^ ^]^ The realm of on‐skin‐based electronics necessitates an elastic substrate that possesses not only soft but also robust mechanical resilience, thereby ensuring a seamless interface with the intricate structures of the skin, particularly within microfluidic collection applications. In this endeavor, we embedded the highly sensitive electrode laser‐engraved graphene (LEG) layer onto a slim adhesive polydimethylsiloxane (PDMS) substrate through the transfer printing method (as illustrated in **Figure** **4**a; Figure S17a, Supporting Information) and subsequently undertook deployment of targeted electrical biosensors (Figure 4b,c; Figure S17, Supporting Information), and then meticulously laminated with a biosponge microfluidic PDMS layer (Figure S17c, Supporting Information) in order to overcome the weak adhesion caused by the small Van der Waals force interaction between the conventional PDMS and PI‐based LEG layer.

Simultaneous monitoring of biofluid analytes and sweat rate is particularly critical for prolonged wearable sensing applications, particularly for individuals contending with diabetes. This demographic is vulnerable to unforeseen fluctuations in perspiration patterns, particularly during instances of hypoglycemic episodes or diabetic neuropathy.^[^ ^45^ ^]^ First, we integrate a sweat rate sensor alongside an enzymatic glucose sensor within a unified sensing unit (the three‐electrode system displayed in Figure 4b). Ordinarily, under the guidance of glucose oxidase, glucose and oxygen are preferentially oxidized to form gluconolactone and hydrogen peroxide (H_2_O_2_) as intermediate products. Given the anticipated low concentrations of the target analytes, the embedded internal polyaniline layer along with Pt nanoparticles, collectively serving as an ion‐to‐electron transducer that acts to amplify the discerning capabilities with regard to the intermediate product H_2_O_2_.^[^ ^46^ ^]^ The liberated free electrons induce a polarization effect on the conductors, consequently yielding a substantial shift in response signals that ultimately enhance sensitivity and also substantiate the concentration of the target glucose. Figure 4d illustrates the chronoamperometric measured sensing response of target glucose encompassing physiological ranges spanning from 0 to 300 µM in PBS‐based analyte solutions with an impressive sensing threshold of 0.28 × 10^−6^ M (LOD). The discerned linear correlation between the current density and the analytical concentrations yielded a sensitivity of 1.07 nA µM^−1^ per cm^2^

The microfluidic channels incorporate vertically sculpted interdigitated electrodes designed in a wheel‐like configuration, serving as a smart sweat rate sensor (Figure 4e). When sweat encapsulated within the channels contacts the initial pair of electrodes, it simultaneously triggers the monitoring process. Intriguingly, as the fluid continuously progresses through the microchannel that encounters a new spoke, a sudden pulse is generated due to an instantaneous non‐Faraday current, resulting in an immediate drop in potential. Each pair of spokes encountered by the advancing fluid generates distinctive pulses, and the recorded time interval between these pulses serves as a rough representation of the sensor signal. By calculating the time intervals between signals and the total number of responses, we can effectively predict and monitor discrete increments in the additional volume of harvested fluid and sweat rate. In contrast, the wearable patch lacking the biosponge (as depicted in Figure S18a, Supporting Information) exhibited a significantly prolonged harvesting time under identical controlled experimental conditions. This observation confirms our prediction that the biosponge‐equipped microfluidic system not only accelerates the harvesting process but also mitigates latency in the sensing response without causing interference. This underlines the pivotal role of the biosponge in optimizing the sensor performance and ensuring prompt and accurate sweat rate monitoring.

Furthermore, we conducted a comprehensive evaluation of alternative arrangements for electrochemical sensors, each aimed at discerning target biomarkers linked with symptomatic manifestations for early diagnosis and predictive analytics. Uric acid (UA) stands as a readily detectable constituent within sweat samples, demonstrating a positive correlation with its presence in serum. Its detectability within sweat holds substantial importance in the realm of early individual diagnosis, particularly as a predictive indicator for the proficient identification of prevalent inflammatory arthritis, notably encompassing conditions such as hyperuricemia or gout.^[^ ^44^ ^]^ The endeavor of constructing a biosensor within a confined active area has invariably been accompanied by challenges. In response, we augmented the sensing sensitivity through the electrodeposition of immobilized gold (Au) nanoparticles and subsequently followed by a secure encapsulation using a polymer characterized by an abundance of amino groups. This enhanced UA sensor exhibited minimal impedance alteration (Figure S18b, Supporting Information) and a distinct oxidation current peak at ≈0.25 V (Figure 4f). Demonstrating sensitivities of 2.94 µA µM^−1^ per cm^2^ at physiological concentrations, this sensor exhibited remarkable selectivity as it remained scarcely affected by other sweat biomarkers, as elucidated in Figure 4f and Figure S17c (Supporting Information). Moreover, the UA response demonstrated repeatable and favorable linear correlations (Figure S18d, Supporting Information), achieving an exceptionally low detection limit, detecting concentrations as low as 0.63 µM. Lactic acid assumes significant prominence as an unavoidable contributor, given its significant impact on sweat pH and its propensity to introduce inaccuracies in UA detection. Thus, simultaneous monitoring of UA and pH within our wearable patch undeniably enhances precision. However, the expansion of the patch's layout to accommodate additional sensors carries the potential to undermine its comfort and suitability, particularly within specialized detection areas like fingertips. A substantial proportion of ammonium sites on the chitosan film surface undergo protonation to form –NH^3+^ within a peracid environment. This protonation process, which entails electron loss, enhances the film's capability to adsorb anions or electrochemically active molecules, thereby heightening sensitivity, particularly in acidic solutions.^[^ ^47^ ^]^ Consequently, the shift in the detection of the UA oxidation peak, accompanied by an amplified current intensity, directly ensues from the decrease in pH within the targeted solution (Figure S18e, Supporting Information). Nonetheless, the fitted curves, which exhibit a favorable linear correlation among the results obtained from differential pulse voltammetry (DPV) as illustrated in Figure S17f (Supporting Information), coupled with the correlation formulas extracted from the peak potential and current intensity of UA oxidation (Figure S18f, Supporting Information), present a noteworthy advantage. This provides an alternative avenue to simultaneously ascertain pH and UA concentration, facilitating the identification of authentic sweat samples while employing a limited count of sensing components and experiencing diminished energy consumption.

An alternative sensor configuration (A variant electrode type displayed in Figure 4b) is the incorporation of an ion‐selective membrane (ISM), notable for its inclusion of ionophores within the PVC matrix, thereby facilitating a discerning and reversible interaction with target ions. This interaction occurs through the Donnan exclusion effect, wherein ions harboring opposing charges experience impediments while facilitating the seamless exchange of the desired target ions. Thus, the ionophores confer ion selectivity to the sensor. Nonetheless, it's important to acknowledge that the PVC matrix may occasionally constrain ion mobility, culminating in a diminished efficiency of ion transportation relative to aqueous solutions. The diminution in transport efficiency is further exacerbated by the limited sample volume available for detection, further compromising the detection performance of the ISM senso.^[^ ^48^ ^]^ To address this, we integrated a polyelectrolyte film between the ISM and substrate, comprising of poly(3,4‐ethylene dioxythiophene): poly(styrene sulfonate) (PEDOT: PSS), functions as an ion‐electron transducer. This mediator adeptly alters the surface potential, thereby heightening the real‐time responsiveness to fluctuations in ion concentration along its interface. This significantly boosts the entire detection system with the efficacy of sensitivity.^[^ ^49^ ^]^ Figure 4g,h illustrate the individual performances of the [Na^+^] and [K^+^] sensors across electrolyte solutions encompassing the standard sweat concentration range for each analyte, spanning from 12.5 to 200 mM and 2 to 32 mM, respectively. The sensors demonstrated a quantifiable alteration in potential signals, showcasing prompt and consistent responses throughout the measurement duration. Importantly, the sensitivities of these sensors are recorded at 33.2 mV and 42.3 mV per decade, close to the Nernstian behavior. For pH detection, the deprotonation at the interface of the electrodeposited polyaniline (PANI) layer serves as a measurable indicator of the overall H^+^ concentration in the bulk solution. Displaying a sensitivity of 55.87 mV pH^−1^, the pH sensor exhibits remarkable reproducibility and establishes a linear correlation within the pH 4–8 range of McIlvaine's buffer (as showcased in Figure 4i). The presence of multiple metabolites coexisting in sweat and manually fabricated sensing layers both contribute to variations in sensor performance. Therefore, it is imperative to conduct tests of selectivity, reproducibility, and stability for each sensor within our combinations. Additionally, evaluating the strain and temperature dependence responses of each sensor highlights its stable usability during routine activities. The recorded sensing performance, as depicted in Figures S19 and S20 (Supporting Information), demonstrates a linear response with minimal fluctuations, showcasing high sensitivity, favorable selectivity, and reproducibility.

When the porous interface integrates with wearable sensors, the rapid diffusion of oxygen molecules, the flux of analyte molecules, and the advective flow rate, which transports analytes onto the sensor surface, become crucial factors in electrochemical sensing and response. In microfluidic electrochemical sensing setups, the absence of flow leads to active analyte consumption on the sensor surface, leading to the expansion of an undesirable analyte depletion zone and causing a delay in the sensing response. Conversely, sensing within a microfluidic chamber with advective flow establishes a continuous analyte supply that prevents the expansion of the depletion zone, ensuring a consistent sensing state.^[^ ^50^ ^]^ Prior studies have effectively proven the utilization of channels as narrow as 10‐micron‐width conduits, adeptly harnessing capillary action to sufficiently draw nanoliters of sweat samples from the skin's surface and channel them onto the sensor.^[^ ^18^ ^]^ Notably, sweat, particularly insensible type, is naturally secreted at an extremely slow rate (just a few nL min^−1^ per cm^2^), even without physical activity. Upon analyzing SEM images of our designed biosponge matrix (Figure 1e–g), it becomes evident that the microchamber, structured hierarchically with micro‐nano pillars, adeptly accommodates the sweat sample. This structural arrangement maintains a steady flux passing through the sensor, preventing the growth of the depletion zone on the sensor surface. In practice, a narrow, elongated channel holds greater capacity but introduces substantial hydraulic resistance to the device. This leads to dynamically fluctuating flow rates that eventually diminish to zero when resistance becomes dominant, inevitably yielding inaccurate biomarker measurements. Integrating a biosponge within the microchannel effectively addresses this by utilizing its superhydrophilic nature to exploit capillary and wettability mechanisms, thereby reducing the device's hydraulic resistance to a negligible level. To evaluate the impact of flow rate on sensor performance within the biosponge‐equipped microfluidic system, a flow rate dependency test was conducted. The noticeable signal fluctuation evident in the glucose sensor's response can be attributed to low flow rates (below 100 nL min^−1^), which hinder the effective mixing and conveyance of compounds within the fluid, resulting in inadequate mass transfer and unstable signal response (Figure S21a,b, Supporting Information). This instability also occurs in conventional microfluidic setups lacking the biosponge. Within a microfluidic system, upon surpassing a flow rate threshold of 100 nL min^−1^, a confluence of adequate convective mixing and uniform dispersion of the substrate within the flowing sample precipitates heightened deposition and adsorption kinetics on the sensor surface (Figure S19c, Supporting Information). Consequently, a more stable sensing response is achieved. Similar flow‐rate dependency tendencies were exhibited by the ISM sensors, whereas the pH sensor encountered minimal influence, as illustrated in Figure S21 (Supporting Information). This could be ascribed to the direct interaction between hydrogen ions [H^+^] and the upper polyaniline stratum, wherein the processes of doping and de‐doping exert negligible perturbations upon mass transfer, thus conserving the sensor's stability and precision in its sensing capabilities. Nevertheless, ion‐selective membranes, fortified with a protective Nafion coating, manifest vulnerability to attenuated transmission rates associated with lower flow velocities. This, in turn, could affect reaction kinetics due to the potential decrease in transmission rate. This relationship warrants consideration when quantifying the concentrations of target analytes during on‐body experimentation, particularly in light of the typically modest sweat secretion rates experienced in everyday circumstances.

### 2.6 Near‐Rest Perspiration and On‐Body Biomarker Validation of Wearable Patch

The microfluidic patch has demonstrated remarkable proficiency in enabling real‐time monitoring of perspiration rate dynamics across a spectrum of activities, spanning from routine physical exertion to prolonged periods of inactivity, as illustrated in Figure 3d. It is important to note that the interplay between various physical activities and internal metabolic can yield synergistic or antagonistic effects on the body's physiological responses. This nuanced interrelation, exemplified by the counteractive influence of food consumption on glucose levels or the modulatory influence of exercise, results in real‐time fluctuations in multiple biomarker concentrations within the body.^[^ ^51^ ^]^ Our fully integrated microfluidic patch, equipped with meticulously designed biosensors, is capable of conducting in‐situ assessments of metabolic biomarker levels in real‐life scenarios.

**Figure 5:**
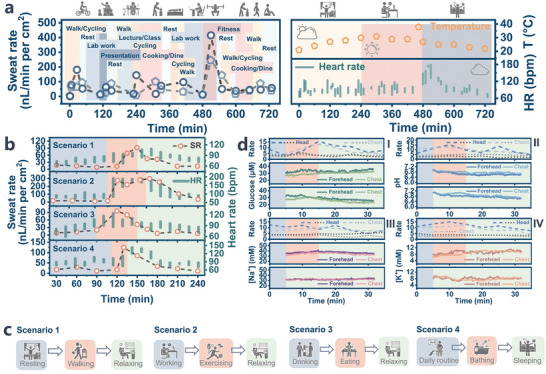
Prolonged personalized sweat assessment during routine activities by utilizing biosponge patch. a) Continuous on‐body sweat‐rate analysis employing biosponge microfluidic system mounted on subject's forehead during performing everyday tasks and capturing dynamic sweat behaviors. b) Regional examination of sweat regulation during various scenarios depicted in c). d) Comprehensive approach involves simultaneous monitoring of target sweat biomarkers and regional dynamic sweat behaviors. Sweat measurement commences following a warm‐up secretion preparation (Blue section). Initiation of sweat biomarkers occurs 15 to 30 min after intake of high‐sugar, high‐calorie diet or moderate exercises (Red section). Green section signifies daily activities such as sitting, walking, relaxing, etc.

Elevated ambient temperatures, coupled with increased relative humidity typical of the summer season, autonomously initiate thermoregulatory sweating mechanisms aimed at regulating core body temperature. This natural response expedites the harvesting of perspiration, significantly reducing the waiting period, even during sedentary or sleeping. For our monitoring, we selected the forehead and chest as the regions for monitoring sweat activity. Simultaneously, we employed a wearable heart rate monitor (Apple watch) to measure heart rate continuously. Leveraging the convenience of optical readout and electrical sweat measurements, ensuring the provision of stable signals, we patiently awaited the secretion of sweat into the sensing positions before commencing long‐term monitoring. The observed results, as depicted in **Figure** **5**a,b, reveal distinct patterns. During sedentary or resting phases, the subject exhibited consistent but relatively low‐rate sweating. However, when engaged in routine activities such as walking, exercising, laboratory schedules, or food intake, the subject transitioned into a state of higher and more fluctuating perspiration, accompanied by increased heart rate. Subsequently, the subject returns to a basal state following relaxation, consistent with findings from prior studies.^[^ ^11^, ^17^, ^39^ ^]^ The interesting thing is the different types of food intake triggered varying intensities of metabolic responses, stimulating food such as spicy or greasy, induced a higher sweating rate along with a more pronounced elevation in heart rate, whereas the ingestion of bland foods elicited a milder response (Figure S22, Supporting Information). Meanwhile, regional sweat loss at a much more aggressive speed represents rapid fluid depletion and electrolyte consumption which could be used to predict whole‐body fluid loss or a warning signal to remind the subject is suffering from dehydration or electrolyte imbalances, especially for the high‐temperature operation or long period of practice and competition.

On one hand, as expected in real‐life scenarios, the relevant biomarkers in sweat exhibited a proportional increase following a period of digestion. In conjunction with the monitoring of sweat rate and heart rate, the biomarker monitoring results for a healthy male subject across two distinct body regions, namely the Forehead and Chest, as depicted in Figure 5c. The sweat glucose sensor revealed a progressive elevation in glucose levels (Figure 5dI) ≈15–20 min after consumption of a sugar‐rich diet, while no such fluctuations were observed in the absence of food intake. The pH value (Figure 5dII) within the sweat exhibited a relatively stable range between 6.4 and 7.1, with minimal fluctuations observed throughout the entire observation period, whereas the lactic acid generated during some vigorous exercise may cause a distinct decrease in sweat pH, but we didn't observe that signal. A similar trend was also recorded in the relatively low concentration of sweat [K^+^] (Figure 5dIII). Conversely, the level of another electrolyte, [Na^+^] (Figure 5dIV) displayed slight variations initially, with the two sampled body spots exhibiting distinct secretion rates. Eventually, [Na^+^] levels stabilized at slightly different concentrations, ≈35.23 mM for the Forehead and 32.67 mM for the Chest region. The concentrations of selected biomarkers obtained from the real‐time response readouts consistently demonstrated reliable readings, closely aligning with the results obtained from conventional laboratory analysis (Figure S23, Supporting Information).^[^ ^24^, ^43^ ^]^ This further validates the accuracy of the biosponge microfluidic platform, enhancing its potential for precise and real‐time health monitoring applications.

On the other hand, the sweat rate demonstrates a strong correlation with variations in heart rate, stimulus responses, and in‐vivo metabolic activity. It tends to increase proportionally with the duration or intensity of physical activity and subsequently returns to lower levels. Stress‐induced feedback during the entire monitoring period, whether caused by consuming spicy foods or engaging in intense dumbbell fitness, is characterized by a sudden surge in sweat rate, accompanied by an elevated heart rate. This elevated and sustained high sweat rate over an extended period suggests a rapid loss of bodily fluids and various electrolytes. While the Forehead and Chest regions exhibited slightly different perspiration rates, both regions displayed a positively correlated secretion rate and [Na^+^] content, as illustrated in Figure 5b,c IV, and Figure S21 (Supporting Information). This consistent trend aligns with existing literature, which indicates that increased electrolyte excretion generally corresponds to higher sweat rates.^[^ ^40^, ^42^, ^52^ ^]^ Conversely, feedback from other routine activities resulted in more moderate fluctuations in the observed indices, serving as a potential baseline for sweat as a viable monitoring mode. However, it is important to note that on‐human sweat measurements revealed intra‐individual variations in sweat generation and collection, as well as inter‐individual variations dependent on body location. Despite these variations, feedback from routine activities and their modest index fluctuations present promising potential for utilizing sweat as a valuable monitoring method.

## 3 Conclusion

We propose a novel wearable patch featuring a biosponge microfluidic device, specifically designed to enhance epidermal fluid sampling, even in minute quantities. Its remarkable superhydrophilic properties enable near‐real‐time perspiration rate sampling. This capability extends beyond merely capturing sensitive sweat induced by external stimuli; it is also adept at capturing minute volumes of insensible sweat secreted by eccrine glands, an area scarcely explored in previous studies. Our preliminary on‐body tests explore the potential correlation between real‐life sweating scenarios and their synergistic or counteracting effects on metabolic responses and external stimuli. Looking ahead, we plan to evolve our patches by integrating scalable flexible electronics and tailored combinations of biomarker sensors on stretchable substrates. This advancement will deepen our understanding of analyte secretion mechanisms and offer guidance on the interpretations of reliably measured concentrations. Additionally, the incorporation of physical sensors enhances the patch's capabilities, enabling simultaneous monitoring of skin and sweat temperatures alongside sweat composition analysis. This integrated system is also amenable to coupling with artificial intelligence‐driven signal processing, allowing our wearable patch to seamlessly adapt to a wide range of personalized health‐monitoring requirements. Thus, it provides a fresh opportunity for comprehensive and tailored well‐being assessments, versatile for addressing a diverse spectrum of individual daily health‐monitoring needs, marking a significant step forward in personalized health technology.

## 4 Experimental Section

### Materials and Reagents

Sodium alginate (SA), calcium sulfate, boric acid (BA), hydrochloric acid, sulfuric acid, and aniline were procured from WAKO. Sodium carboxymethylcellulose (CMC) was obtained from Toronto Research Chemicals. Dimethyl sulfoxide (DMSO), hydrogen hexachloroplatinate (IV), chitosan, polyvinyl alcohol (PVA, Mw 89000–98000), graphene powder (electrical conductivity > 103 S m^−1^), Nafion 117, polyvinyl butyral (PVB), silver/silver chloride (60/40) ink, and graphene/PEDOT: PSS hybrid ink were sourced from Sigma–Aldrich. These reagents were used as received without further purification. Solutions for the study were prepared using Milli‐Q water (18.25 MΩ cm^−1^).

### Preparation of Mycelial‐Chitin (M‐Chitin)

Mycelial‐chitin (M‐Chitin) was extracted from the fungal mycelium of *Aspergillus niger* strain (CICC 2487) cultured on a potato dextrose agar slant. Initially, the harvested raw mycelium underwent a 20 min steaming process and was then thoroughly washed with Milli‐Q water.^[^ ^53^ ^]^ Subsequently, a household blender was employed to crush the mycelium cells, and the resulting slurry underwent filtration to eliminate intracellular material. The residual slurry, constituting the cell wall, underwent sequential immersion in 2.0 M HCl for 48 h and then 4.0 wt.% NaOH for an additional 48 h. The solid residue (post‐Lyophilization) underwent bleaching treatment by immersion in a 0.5 wt.% NaClO_2_ solution (pH 5.0, acetic acid) for 2 h at 70 °C. The purified Mycelial‐chitin was then carefully washed with Milli‐Q water and subsequently stored at room temperature following freeze‐drying.

### Preparation of Deacetylated Mycelial Chitin (DM‐Chitin)

To mitigate the adverse effects caused by the abundant liquid content in undried chitin samples, which hampers the deprotonation of amides and hydroxyl groups in DMSO/KOH,^[^ ^54^ ^]^ freeze‐dried raw mycelial chitin samples were employed for extraction and further modifications. The dry M‐chitin was immersed in DMSO saturated with 1.0 mg mL^−1^ KOH under vigorous stirring for 24 h. The product was then centrifugally washed with ethanol to remove unreacted solvents and dispersed in a 30 wt.% NaOH aqueous solution by magnetic stirring at 80 °C for 3 h. Subsequent thorough washing with Milli‐Q water, until the product reached a neutral pH and stored at room temperature after freeze‐drying. This process yielded partially deacetylated chitin (DM‐chitin), which was further washed with distilled water to neutral pH and dried at room temperature.

### Preparation of Fiber‐Like Deacetylation Mycelial Chitin (FDM‐chitin)

The Fiber‐like Deacetylation Mycelial chitin (FDM‐Chitin) was produced through a mechanical method. Initially, the prepared DM‐Chitin was redispersed in an acetic acid solution under vigorous stirring, and the suspension underwent homogenization using a high‐speed blender. Subsequently, the fine suspension was neutralized to a pH of 10 using NaOH, resulting in the precipitation of flocculent FDM‐Chitin, which was obtained after thorough washing with Milli‐Q water. The freeze‐dried and purified FDM‐Chitin was then stored at 4.0 °C for future use. The synthesis method for carboxymethyl FDM‐Chitin (CFDM‐Chitin) followed the procedures outlined in previous literature.^[^ ^55^ ^]^

### Preparation of Precursor Raw Gel

The precursor raw gel was prepared through sequential mixing, with each gel being combined in the designed ratio and vigorously stirred until homogenized. Initially, specific stoichiometric quantities of 1.0 wt.% CMC and 1.0 wt.% SA were mixed in a homogenized dispersion containing 1.0 wt.% CFDM‐Chitin under ultrasonic action, forming Gel A. Simultaneously, Gel B was created by adding 5.0 g of PVA powder to 45.0 g of Milli‐Q water, incubated for over 8 hours in an oil bath maintained at 95 °C. Subsequently, a small amount of BA was introduced into the Gel A: Gel B dispersion at a mass ratio of 8:2, and the mixture was stirred using a homogenizer. The precursor raw gel was achieved after defoaming.

### Fabrication of Microfluidics Biosponge

The fabrication of the biosponge microfluidic patch involved a meticulous process, beginning with the raw gel slurry. This slurry was initially injected into molds and left at −75 °C overnight. Subsequently, it was quickly transferred to a lyophilizer and dried for two days, resulting in a semi‐biosponge with the first polymerized internal skeleton. Following this, the semi‐biosponge underwent a gentle soak in a 4.0 wt.% CaSO_4_ solution for a minimum of 12 h, with two solution exchanges during this period. After immersion in Milli‐Q water to eliminate any unreacted residue, the hierarchical internal skeleton structure of the biosponge was synthesized, followed by deep freezing and lyophilization procedures. Lastly, a simple thermal annealing at 65 °C for 30 min was performed to prevent the collapse of the internal hierarchical structure during long‐term storage.

### Fabrication of Epidermal Biosponge Microfluidic Patch

The microfluidic patches, each with different functional layouts, were fabricated using standard soft‐lithography techniques. The concept with designed pattern was constructed from AutoCAD to a photomask, followed by spin‐coating on a glass wafer with photoresist (KMPR 1010, Kayaku Advanced Materials Inc., formerly MicroChem Corp., USA) at 800 rpm, then baking (65 °C for 15 min, 90 °C for 25 min) until a desired photolithographic patterning was created by exposure to UV light (150 s) through a photomask and another round of baking (65 °C for 5 min, 90 °C for 10 min). Wet etching (KMPR 1010) removed the unexposed regions and yielded a final replication mold (cleaned with isopropyl alcohol and quickly dried with N_2_). Briefly, casting PDMS (mixing ratio 15:1) against lithographically replication molds, subsequently, formed a film flexible elastomer (control the height less than 400 µm) with features of invaginated microchannel on its bottom surface. Specifically, microchannels equipped with biosponge assembly processes began with completing cast of the precursor raw gel into microchannel (blade coating or using syringe) and followed by the subsequent procedure including deep freezing, lyophilization, and cross‐linking. A uniform bottom PDMS layer with 200 µm height was similarly yielded on a glass wafer. Prior to bonding with the upper biosponge‐equipped pattern patch, an oxygen plasma surface treatment (Basic Plasma Research Kit BP‐1, SAMCO Inc., USA) at a power of 90 W, 0.2 mtorr for 2 min, to enhance adhesion. A mechanical punch was used to create an inlet and an air vent. The separate fluidic layer was then mounted, followed by strong covalent bonding through baking in an oven (ProtoLaser R, LPKF Laser & Electronics AG, German) at 70 °C for ≈10 min to complete the assembly of biosponge microfluidic patch.

### Sensor Fabrication and Characterization

The process for generating electrodes onto a thin PDMS layer commenced with a mass‐producible laser‐engraved graphene (LEG) electrode on a 100 µm thick PI film (DuPont) with a 50 W CO_2_ laser cutter (Rayjet 50, Trotec Laser, Inc., USA) and transprint the electrode onto slim PDMS film following reported protocols with a slight modification.^[^ ^28^, ^56^ ^]^ The concentrated CO_2_ laser beam generates an extremely high heat of more than 2500 °C, which could break chemical bonds in the PI network and re‐organize into carbon atoms, resulting in sheets of graphene structures. Raster mode engraving was employed with optimized parameters of laser power, speed, frequency, pulses/dot, and line spacing set at 5.0%, 10 000 Hz, 15.0%, and 1000 points per inch (PPI), respectively, with three‐time scans. Simultaneously, a mixture of PDMS precursor and catalyst (at a 15:1 ratio) was spin‐coated onto a glass wafer at 1000 rpm for 10 s and incubated at 60 °C for 15 min. The PI film with the LEG electrode was then inverted onto the viscous PDMS and firmly fixed the roots of patterned LEG electrodes and completed the incubation at 80 °C for 30 min to ensure electrode adherence and electrical conductivity retention.

Functionalized sensors and Electrochemical characterizations required an electrochemical workstation (PGSTAT128N, Metrohm Autolab, Utrecht, Netherlands) and performed at room temperature (25 °C). The glucose sensor was prepared by initially electrodeposited Polyaniline (PANI) onto the as‐prepared LEG electrode at 0.75 V versus Ag/AgCl in a 0.5 M/1.0 M aniline/H_2_SO_4_ solution for 20 s. Next, to improve the sensitivity, subsequent electrodeposition was performed at 0.5 and −0.7 V versus Ag/AgCl for both alternating 10 s with 50 cycles (0.001 M/0.1 M, H_2_PtCl_6_/KCl). After that, drop‐casted the mixed solution (40 mg mL^−1^ glucose oxidase, 1.0 wt.% chitosan, 2.0 mg mL^−1^ graphene) onto the resulting electrode, then coated with another Nafion film (0.5 wt.% Nafion) and immersed into glutaraldehyde solution (2.0 wt.%) for 30 min. Finally, the achieved glucose sensor was stored at 4 °C overnight. To obtain the high‐sensitivity UA sensor, Au electrodeposition on the working electrode was adapted from a previous report.^[^ ^57^ ^]^ An Au electrodeposition solution (40 mM/0.1 M, HAuCl_4_/HClO_4_) was adopted at −0.08 V versus Ag/AgCl for 120 s. Second, 1.0 wt.% chitosan solution was dip‐coated and immersed into glutaraldehyde solution (2.0 wt.%) for 15 min, followed by incubation for at least 24 h. The electro‐polymerization of aniline onto the LEG operation electrode with a solution containing 0.1 M aniline in 1.0 M HCl by cyclic voltammetry from −0.2 to 1.0 V (vs Ag/AgCl) at a scan rate of 100 mV s^−1^ for 25 times to generate a PANI layer which was applied for pH sensor. The ion‐selective electrodes were prepared via incubation through drop‐casting as follows: [Na^+^] selective cocktail composed of a mixture formula including 33.0 mg PVC with high molecular weight, 1.0 mg sodium ionophore X, 0.77 mg NaTFPB (sodium tetrakis[3,5‐bis(trifluoromethyl) phenyl]borate ion exchanger), and 66.0 mg DOS (bis(2‐ethylhexyl) sebacate) in 660.0 µl THF (tetrahydrofuran). [K^+^] selective cocktail composed of a mixture formula including 33.0 mg PVC, 0.5 mg KTCB (potassium tetrakis(4‐chlorophenyl)borate), 64.5 mg DOS, and 2.0 mg valinomycin, in 660.0 µl THF.^[^ ^58^ ^]^ To minimize potential drift, a commercial graphene/PEDOT: PSS hybrid ink was drop‐casted onto the LEG electrode and dried overnight, before drying the ion‐selective cocktail layer. A leaching‐prevent layer was subsequently finalized by drop‐casting a polyurethane resin (dissolved 0.5 wt.% in THF). A commercial Ag/AgCl ink was used to fabricate the reference electrode.

### On‐Body Sweat Analysis

The on‐body trial studies were conducted following protocols approved by Research Ethics Committee of the Institute of Pure and Applied Sciences, University of Tsukuba (2023‐06). Healthy young adult volunteers of both genders were randomly recruited through a campus flyers recruitment and provided informed consent before participation. Ethanol swabs and gauze were used to wipe the targeted body locations of volunteers before applying the microfluidic patches and absorbent pads. A commercial wearable device (Apple Watch 8; Apple, CA, USA) was used for heart rate measurement and recording. Sweat composition data were measured by using an electrochemical workstation (PGSTAT128N, Metrohm Autolab). A smartphone camera (iPhone 13 Pro; Apple, CA, USA) captured optical photographs of the devices after separate mounted. An IR camera (A615; FLIR) captured thermal infrared images and measured heat generation from local skin surfaces and microfluidic patches. To guarantee the mounted patches were firmly fixed and stayed in the investigational areas without migration or detachment during the vitro‐body trial, medical adhesive tapes (Single‐Sided Tape ST‐279 and Double‐Sided Tape ST‐502 from Nitto Denko Corporation) were laminated between the skin and the patch and top sealed the patch. A conventional procedure (weighing and laboratory‐based analyses) that applied absorbing pads (Webril Handi‐Pad) as performance compared to the microfluidic patch. Briefly, the primarily designed trial studies were to assess the feasibility of utilizing microfluidic patches in practical scenarios, encompassing controlled and uncontrolled environmental settings, as well as compatible sweat analysis during sedentary, routine, and daily activities. Volunteers were required to wear loose and casual clothing with exposure to large skin surfaces. The indoor trial was conducted at controlled condition of 23–26 °C under the relative humidity of 45%–60%. The trial involved the uncontrolled environment, the outdoor temperature and relative humidity data sourced from the Weather App. Whole‐body sweat loss was measured using a commercial body scale before and after physical activities such as jogging.

### Statistical Analysis

All presented data were collected from separate measurements without additional processing, except for the fitting strategy used to calculate the correlation coefficient. Figures were analyzed and plotted using ORIGINPRO 2023b software. The correlation coefficient, represented as the goodness of fit (R‐squared, *R^2^*), was included in the figure legends. *R^2^* values were computed using Origin software. Data were presented as average values (*n* = 3) and standard deviation (SD), unless otherwise specified in the figure caption.

## Conflict of Interest

The authors declare no conflict of interest.

## Author Contributions

H.L.D. conceptualized the biosponge microfluidic system; H.L.D. and H.Y. preprocessed and synthesized the biosponge; H.L.D. and H.Y. conducted experiments; H.L.D. carried out data analysis and interpretation; H.L.D. drafted the manuscript; H.L.D., H.Y., and T.S. revised the manuscript. The manuscript was written by H.L.D. and with assistance from all authors.

## Supporting information

Supporting Information

Supplemental Movie 1

Supplemental Movie 2

Supplemental Movie 3

## Data Availability

The data that support the findings of this study are available from the corresponding author upon reasonable request.
