# Supplementary material for: Nature‐Inspired Superhydrophilic Biosponge as Structural Beneficial Platform for Sweating Analysis Patch
Source: Adv Sci (Weinh). 2024 Jun 13;11(30):2401947. doi: 10.1002/advs.202401947 (PMC11321618; doi:10.1002/advs.202401947)
Supplement: Supplementary file 1 — Supporting Information [file ADVS-11-2401947-s002.docx]

Supplementary Materials for

**Nature-Inspired Superhydrophilic Biosponge as a Structural Beneficial Platform for Sweating Analysis Patch**

Hanlin Ding ^1*^, Hao^1^ Yang, and Tsujimura Seiya^1*^

Division of Material Science, Faculty of Pure and Applied Science, University of Tsukuba, 1-1-1, Tennodai, Tsukuba, Ibaraki, 305-5358, Japan
E-mail: jellyfishscavenger@hotmail.com, seiya@ims.tsukuba.ac.jp

**This PDF file includes:**

Supplementary Text

Figs. S1 to S23

Tables S1

References (1 to 6)

**Other Supplementary Materials for this manuscript include the following:**

Movies S1 to S3

Supplementary Text

Device Design Rationale and Theoretical Pressure Distribution

The strategy for neutralizing hydraulic pressure loss in the inlet well and managing hydraulic resistance in the extended channel involves the incorporation of a porous biosponge into the conventional microfluidic design. Under the assumed equilibrium condition, eccrine glands serve as autonomous pumps, consistently generating a uniform volumetric fluid at a low flow rate (*Q_sweat_*), thereby maintaining a constant fluid pressure (*P_sweat_*). This fluid is directed into a microfluidic system, introducing hydraulic resistance (*R_device_*, where *R_device_* = *R_inlet_* + *R_channel_*) due to its suspension over the eccrine glands, inducing a pressure drop (*P*_loss_, where *P_loss_* = *R_device_* x *Q_sweat_*). The extended channel, while enhancing harvesting capacity, contributes to augmented hydraulic resistance. Nevertheless, the pumping mechanism necessitates that the secretory pressure of eccrine glands (*P_sweat_*) surpass the driving pressure to sustain the microfluidic system. Consequently, as the fluid from the glands reaches the skin surface, Laplace pressure becomes negligible, and the confined laminated structure impedes evaporation. The tail outlet maintains equilibrium in internal pressure, resulting in minimal atmospheric pressure. This theoretical bi-layer design comprises two pivotal parameters: the eccrine glands layer with a consistent flow rate (*Q_sweat_*) and the harvesting layer with fluid resistance (*P_loss_*).

External stimuli, such as chemical stimuli, can exert significant effects on the hydraulic capability of sweat glands, generating impressive hydrostatic pressures above 72 kN/m^2^ in-vivo, while exercise or sauna-induced sweat yields pressures around 2.5 kPa.^[1-2]^ According to *van't Hoff's law*, the osmolality gradient is linked to the secretion rate, and sweat rate during routine activities exhibits temporal fluctuations. The microfluidic patch fully covering the sweat gland, coupled with a central inlet collection zone preventing evaporation, ensures complete wetting and hydration of the skin surface, rendering the impact of the estimated average secretion speed on pressure negligible. Furthermore, *van't Hoff's* law can be applied to estimate the pressure generated in the gland:^[2]^

*P* = *σRTΔC*

where *P* denotes the change in secret pressure, *σ* and *R* represent the osmotic reflection coefficient and gas constant under an assumed ideal scenario, *T* is the body temperature, and *ΔC* signifies the osmolality gradient.

The pressure drop calculation for the porous structure was additionally compared with the secretory pressure. The exceptional superhydrophilic nature of the biosponge eliminates Laplace pressure contribution to the pressure drop in the inlet well. Upon contact with the biosponge, the fluid readily permeates its internal structure, rendering the pressure drop in the inlet well (*P_well_*) exclusively ascribed to the hydraulic pressure drop induced by the well height, expressed as:

*R_inlet_* = *µH*/*kA* ≈ 1.67 x 10^11^ Pa-s/m^3^

Considering the only laminar flow effect in the porous structure, the permeability (*k*) of porous media can be determined from the integral form of *Darcy’s law* (*Q*= *kAΔP*/*µL*). The hydraulic resistance in the microchannel (*R_channel_*) is expressed by

*R_channel_* = (*128µL*/*W^4^*) x (1/*ε*) ≈ 2.54 x 10^12^ Pa-s/m^3^

where *H* can be estimated for a cylinder corresponding to either inlet well or thickness of biosponge, *L* represents the total length of the porous microchannel, *W* denotes the channel width, *μ* is the viscosity of sweat, considering a 99% water content.^[3]^ *ε* represents the porosity of biosponge.


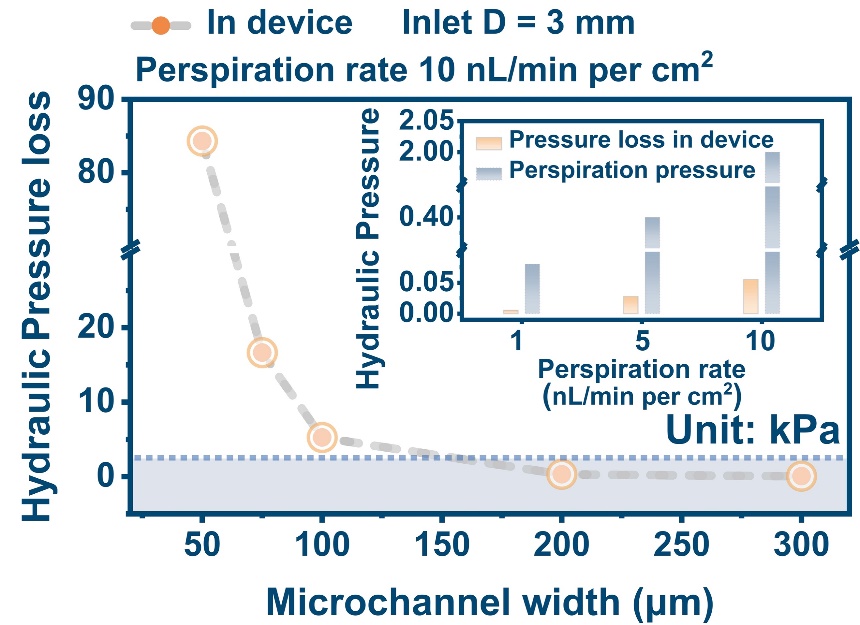


The secretory pressures of sweat glands exhibit variability in response to external stimuli. A conservative estimate, exemplified by a pressure of 2.0 kPa, representative of a high secretion rate ranging from 5-10 nL/min per gland and typical densities of 100 glands per cm^2^, has been considered.^[4-5]^ When comparing this estimated sweat gland secretory pressure with hydraulic pressure drops within our device's designated dimensions, the total hydraulic resistance is found to be 2.71 x 10^12^ in this ideal scenario. Notably, the extended channel with confined dimensions contributes more resistance than the inlet well. However, under specified dimensions and utilizing the biosponge microchannel, our device not only possesses ample harvesting capacity but also circumvents excessive hydraulic pressure losses across a broad spectrum of natural sweat secretion and flow rates. This design ensures that the eccrine gland's ultrastructure functions as an effective pump, delivering sufficient pressure to facilitate the swift conveyance of sweat into a device of these dimensions.


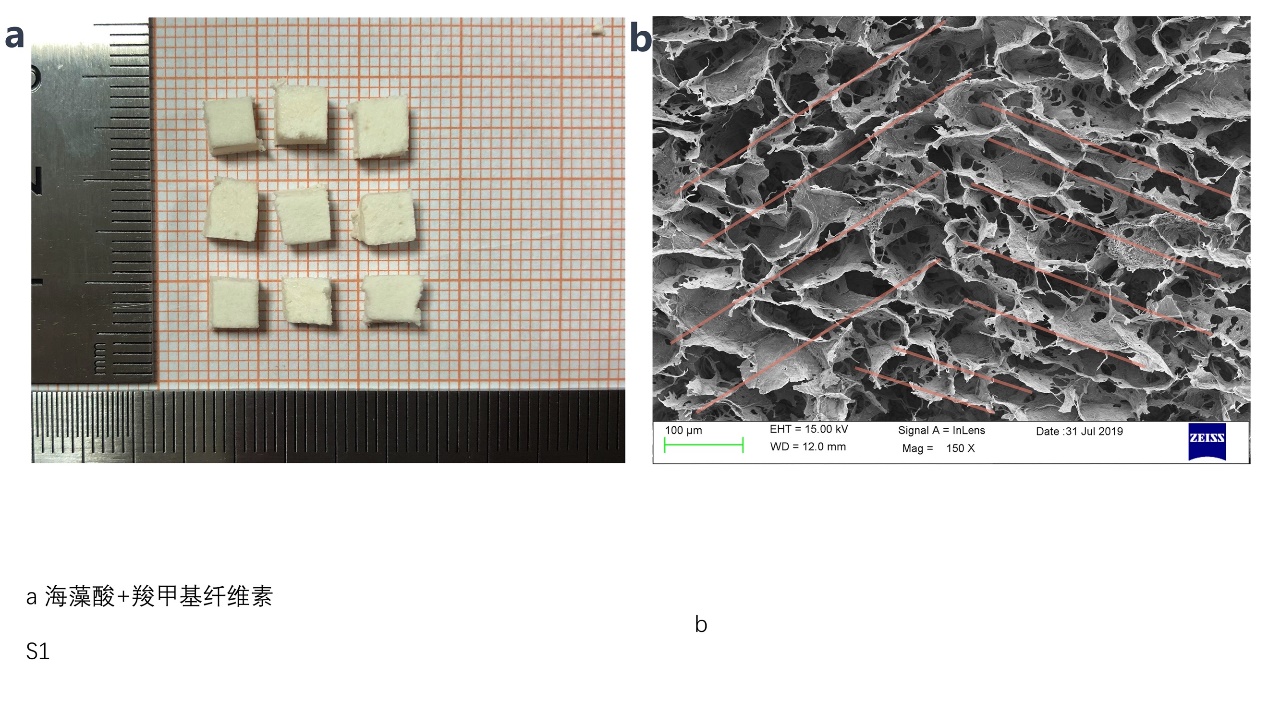


Figure S1. The common pristine sponge and its internal skeletal characterization.

(a) The achieved sponge is fashioned using a double network composed of alginate and carboxymethylcellulose, employing a lyophilization process for its formation. (b) The scanning electronic microscope (SEM) image of internal porous morphologies exhibit distinct lamellar skeletons, which constitute the fundamental structural framework.

Type or paste caption here. Create a page break and paste in the Figure above the caption.


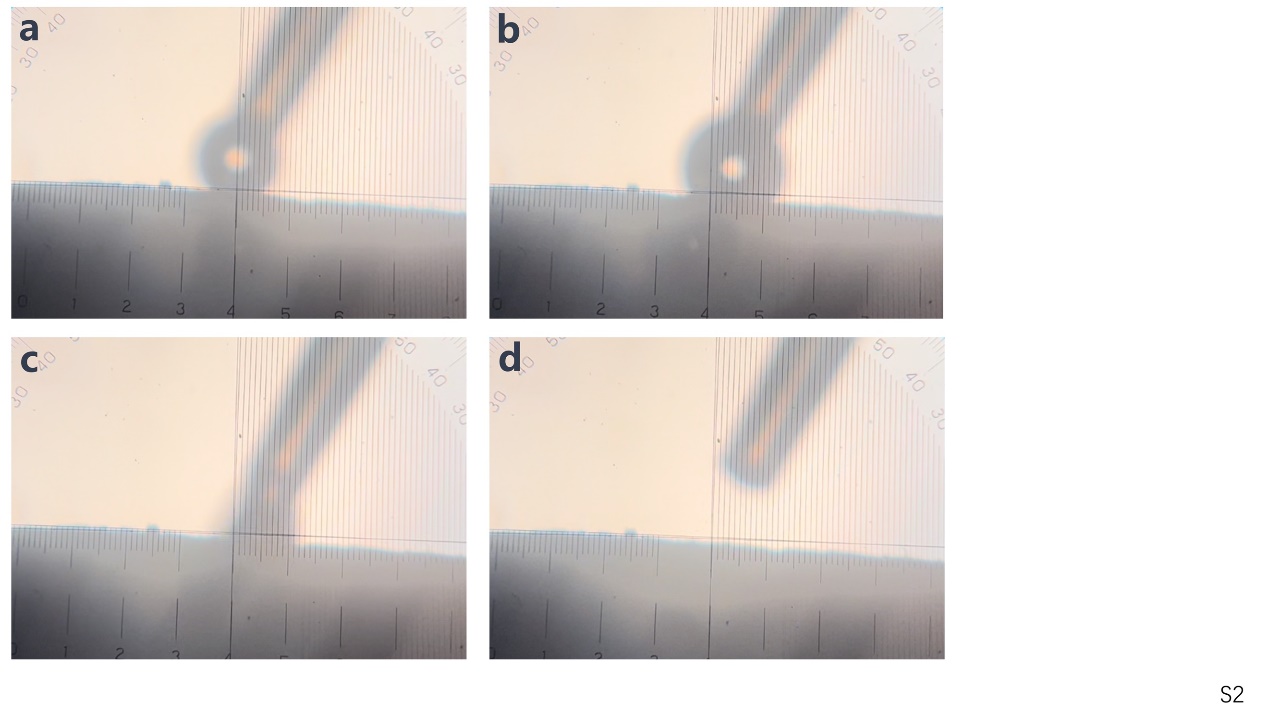


Figure. S2. The superhydrophilic characterization.

A series of images illustrating the progression resembling a liquid droplet predatory-like absorption process on the biosponge surface. The instant absence of liquid residue confirms its remarkable superhydrophilic property.


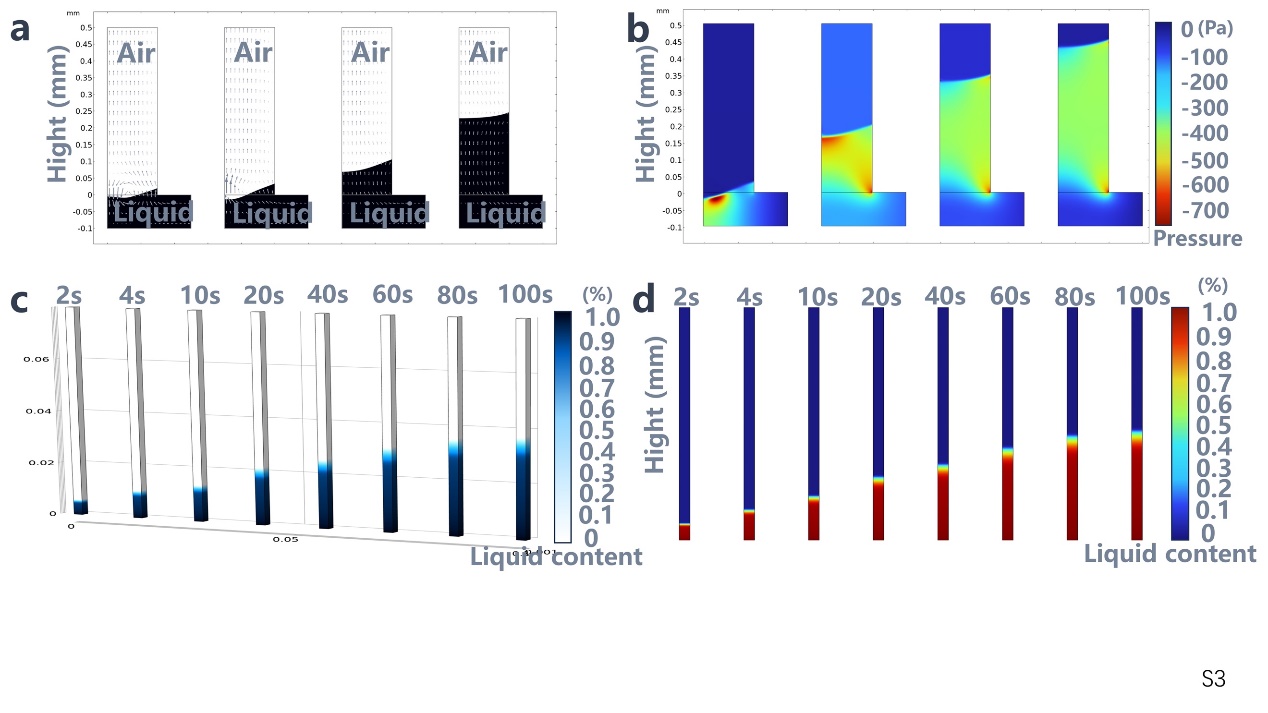


Figure. S3. The COMSOL Multiphysics simulation on the progressive self-climbing of liquid within a slim strip biosponge.

(a) The initial interaction occurs between the fluid interface and the bottom of the biosponge. The surface tension, resulting from the surface curvature, commences the upward movement of water through the slender biosponge column. Due to the instantaneous initiation, the surface experiences slight oscillations during the ascent. (b) Initial pressure jumps at the fluid interface, reaching approximately 300 to 400 Pa. This increase is induced by surface tension and compels the remaining liquid and air within the porous structure to ascend through the narrow biosponge column. (c, d) Capillary motion and surface wettability drive the self-climbing of liquid within the biosponge at various time scales, ranging from 2s to 100s.


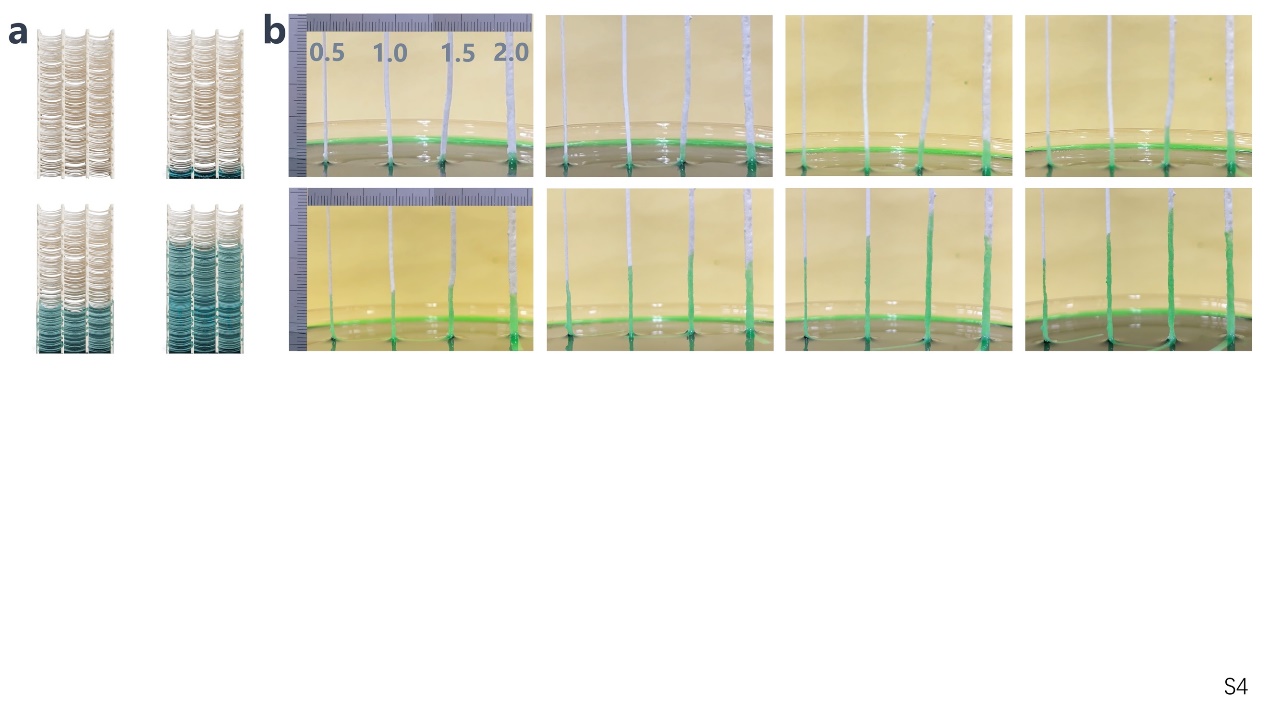


Figure. S4. Alterations in the liquid absorption behavior of biosponge strips, partially immersed in a liquid infused with green dye, manifest changes in their height.

(a) Frontal perspective displaying the progression of self-pumping within tubular fluidic structures. (b) This visualization depicts the time and dimension-dependent saturation of liquid in the self-pumping process within multiscale, vertically aligned biosponge structures.


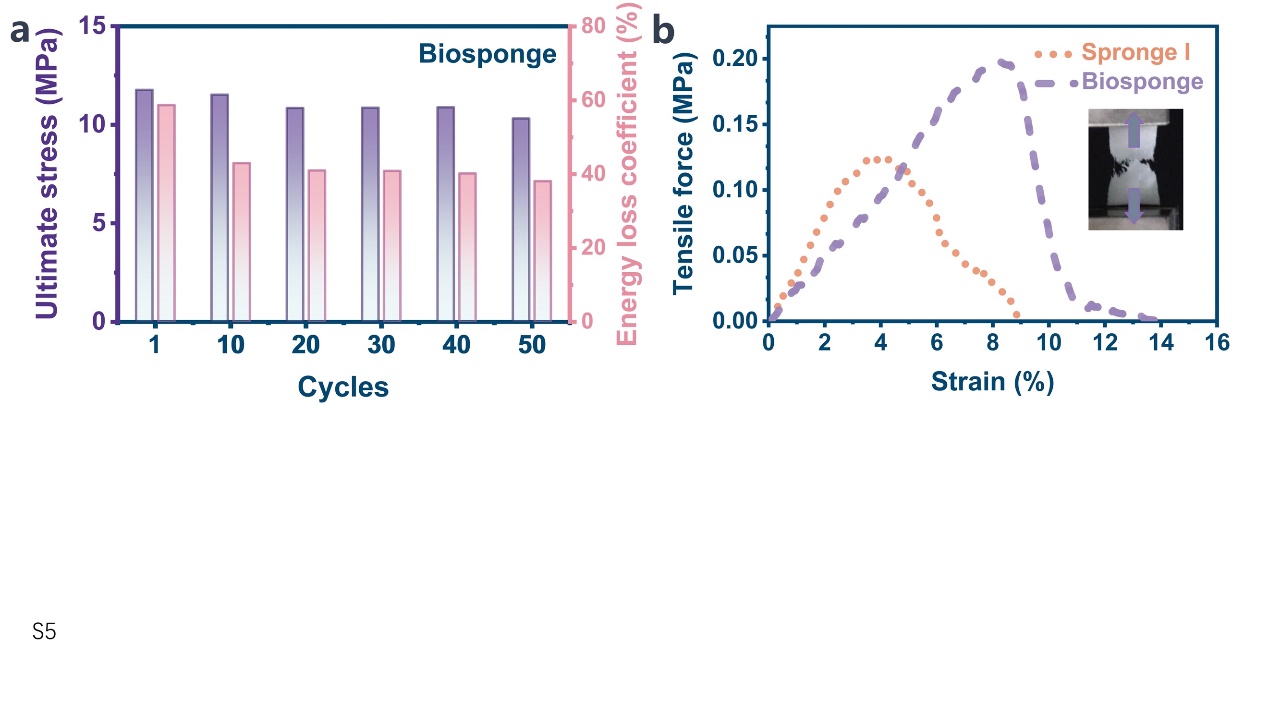


Figure. S5. Mechanical evaluation of biosponge performance.

(a) The ultimate stress at 40% strain and the stress energy loss coefficient curves during 50 compression cycles of the biosponge. (b) The tensile force-strain curve of the mechanically reinforced biosponge (cylindrical with a 10 mm diameter).


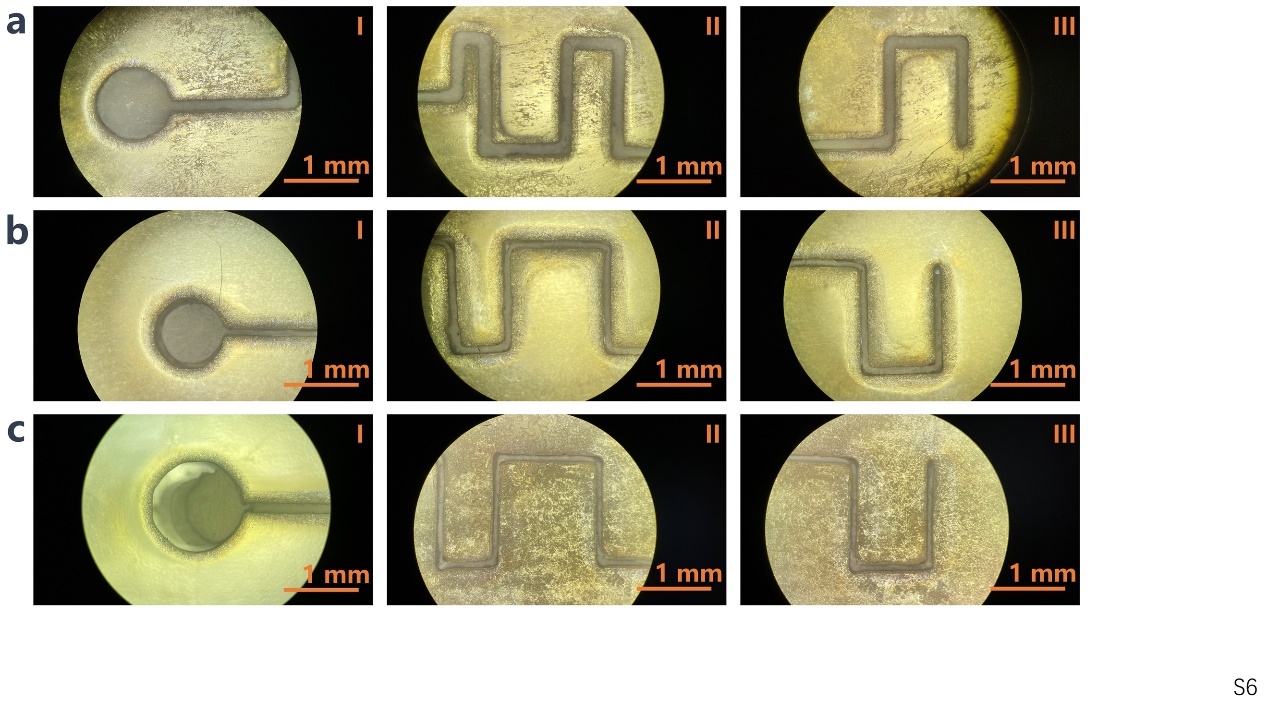


Figure. S6. Evaluating the structural integrity of biosponge microfluidics.

(a, b) The minimal volume shrinkage of the modified biosponge within the multi-shaped and dimensional microchannels after undergoing two lyophilization treatments while preserving its structural integrity. (c) Significant volume shrinkage and structural failure in Sponge I after the last lyophilization cycle.


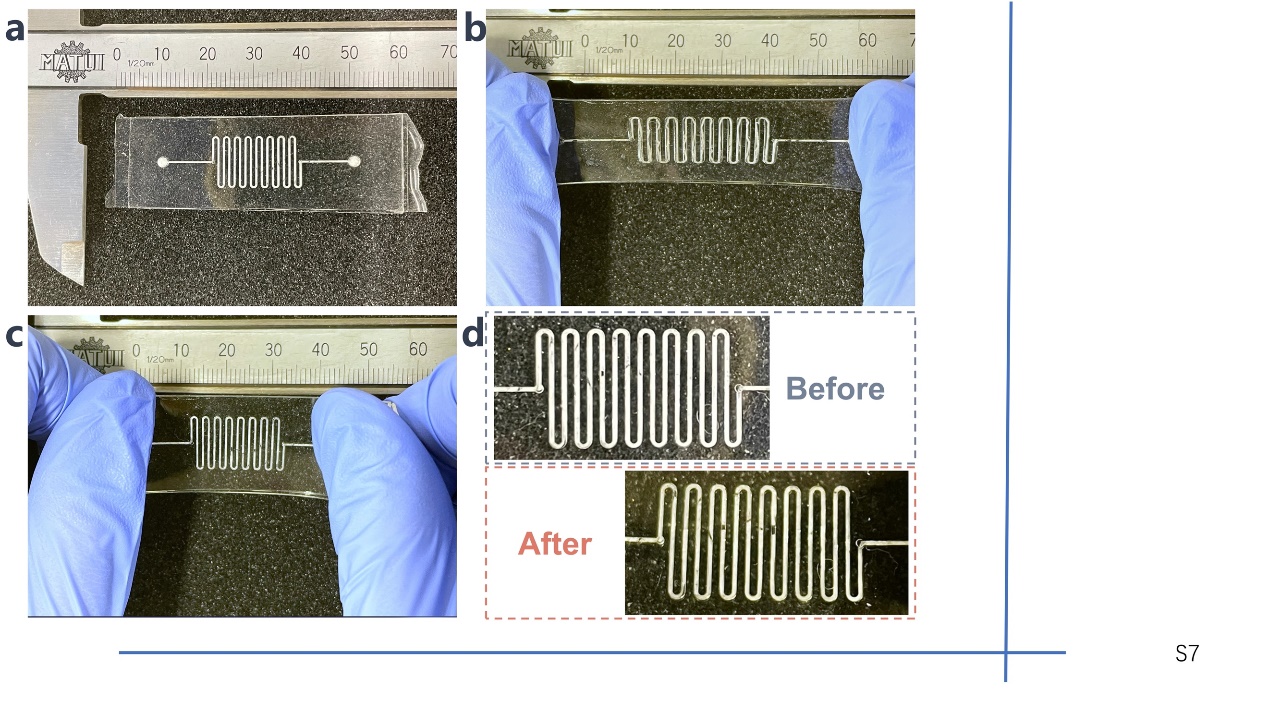


Figure. S7. Assessment of Sponge I microfluidics integrity.

(a, b) The minimal volume shrinkage of the modified biosponge within the multi-shaped and dimensional microchannels after undergoing two lyophilization treatments while preserving its structural integrity. (c) Significant volume shrinkage and structural failure in Sponge I after the last lyophilization cycle.


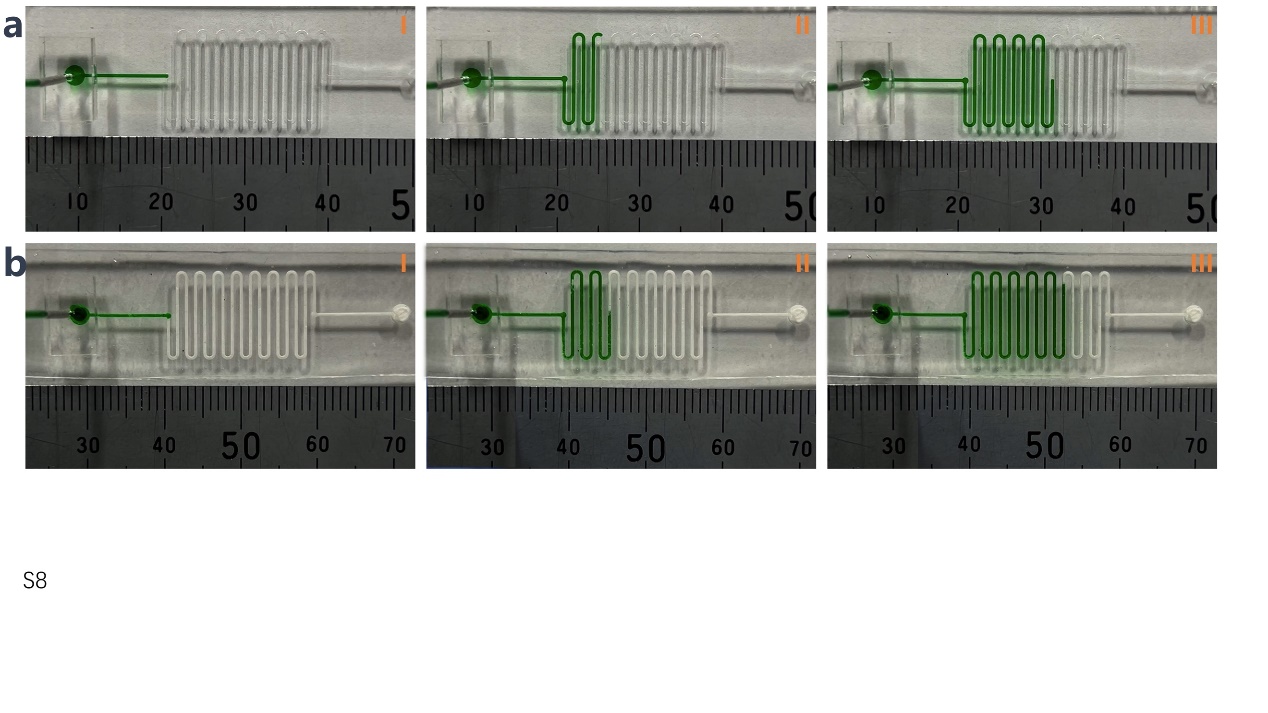


Figure. S8. The comparison of liquid harvesting speed.

(a) The channel without biosponge as a control, (b) biosponge-equipped microfluidics.


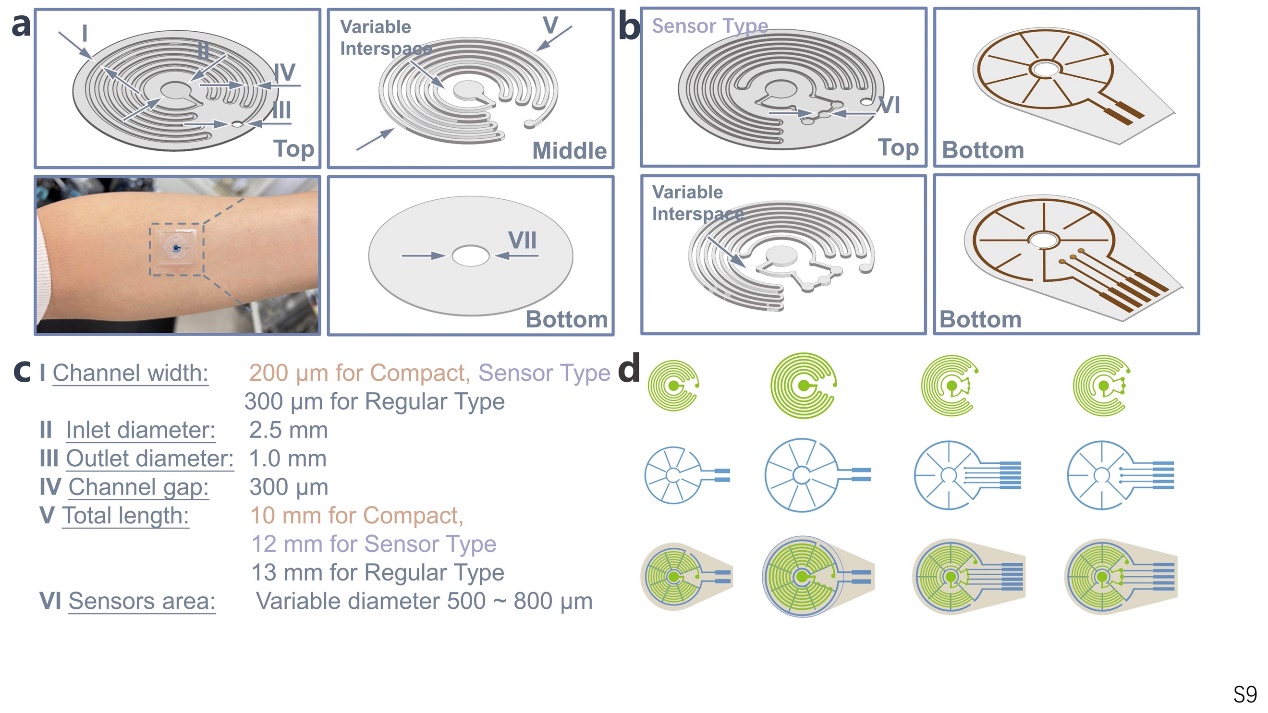


Figure. S9. The expanded geometric parameters and dimensions are elucidated for the disassembled microfluidic patch in its various configurations.


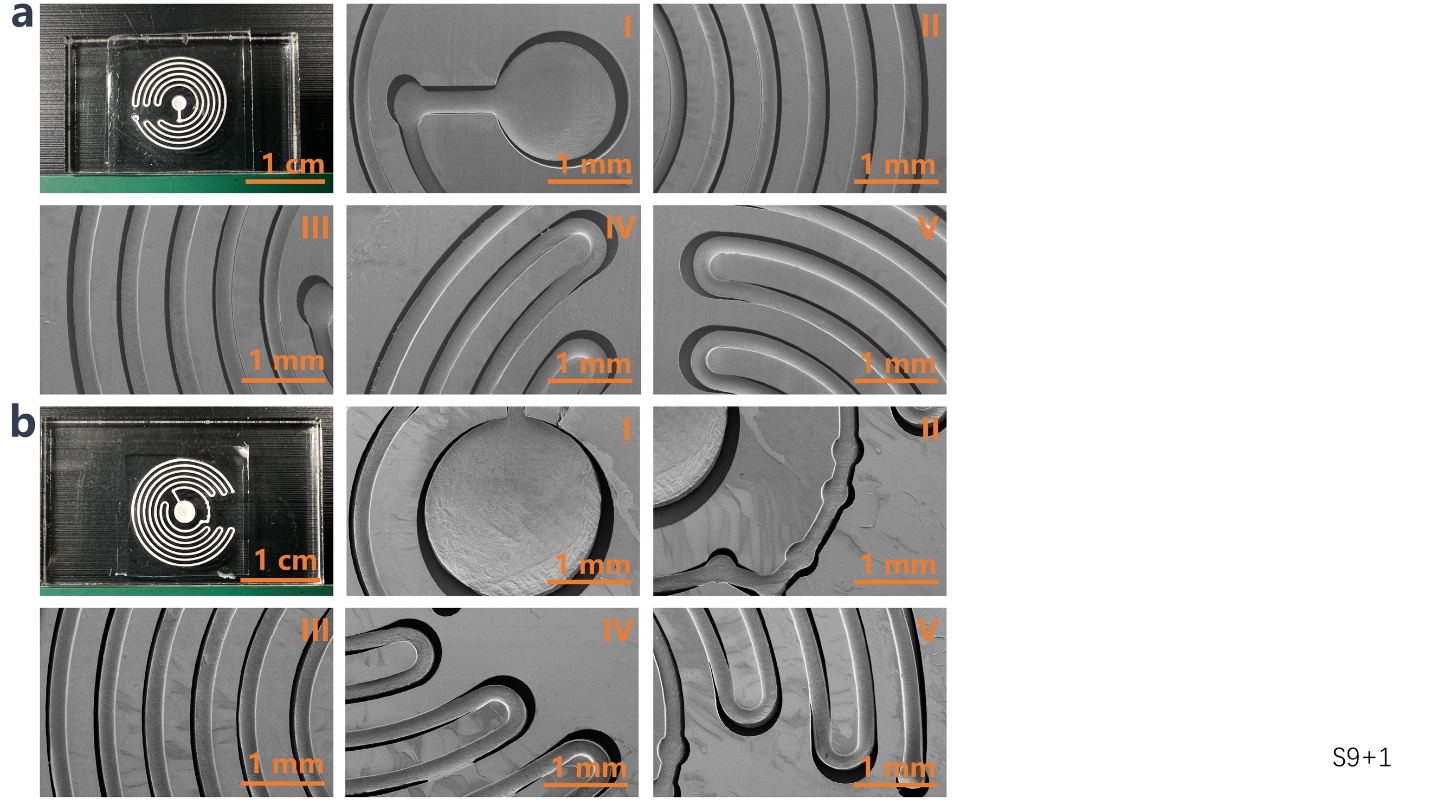


Figure. S10. Scanning electronic microscope (SEM) images reveal the structural uniformity of biosponge microfluidics.

(a, b) The biosponge within the two types of microchannel layouts after undergoing two lyophilization treatments while preserving its structural uniformity.


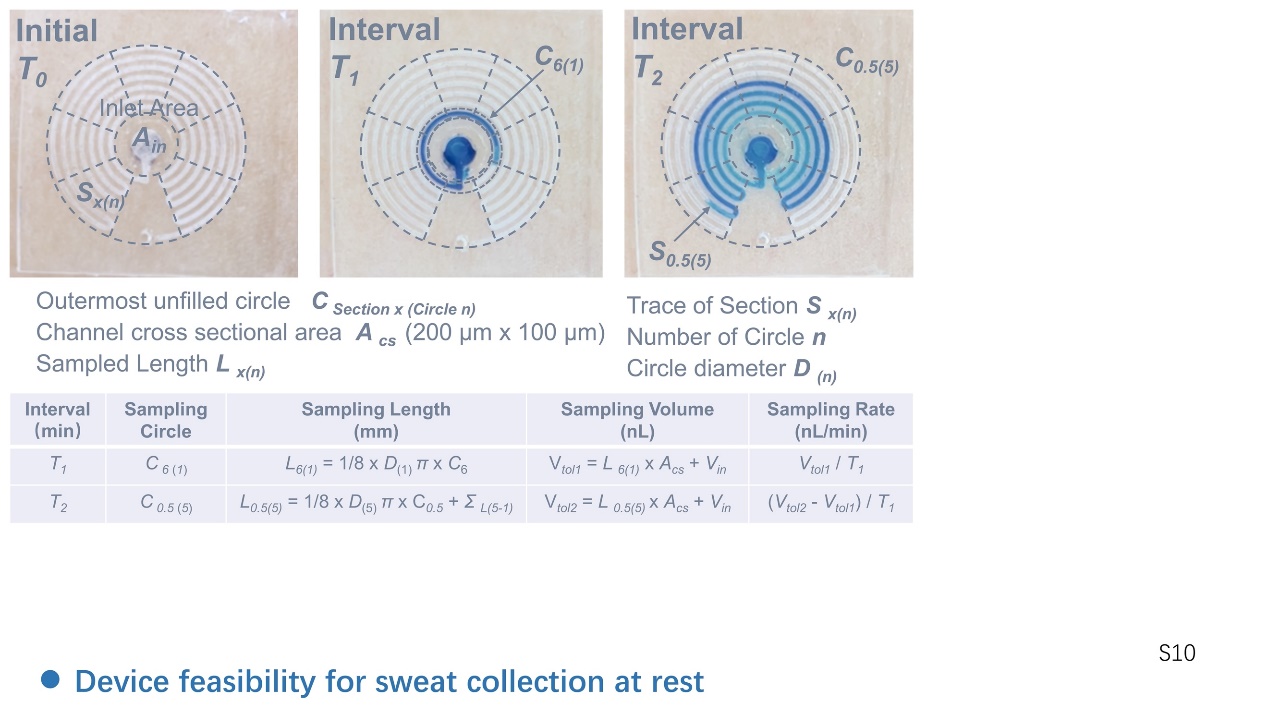


Figure. S11. Perspiration rate quantification is achieved through sweat footprint measurement.

Perspiration rate quantification is achieved through sweat footprint measurement. The blue-dyed biosponge traces the path, aiding in the determination of the channel length occupied by harvested sweat. Essentially, the collection zone is subdivided into eight units, demarcated by dashed gray lines, or more as required, each customized with specific dimensions. Optical images, captured at defined time intervals, serve as inputs for scaling and subsequent conversion into a real-time perspiration rate index through dedicated area trace processing software.


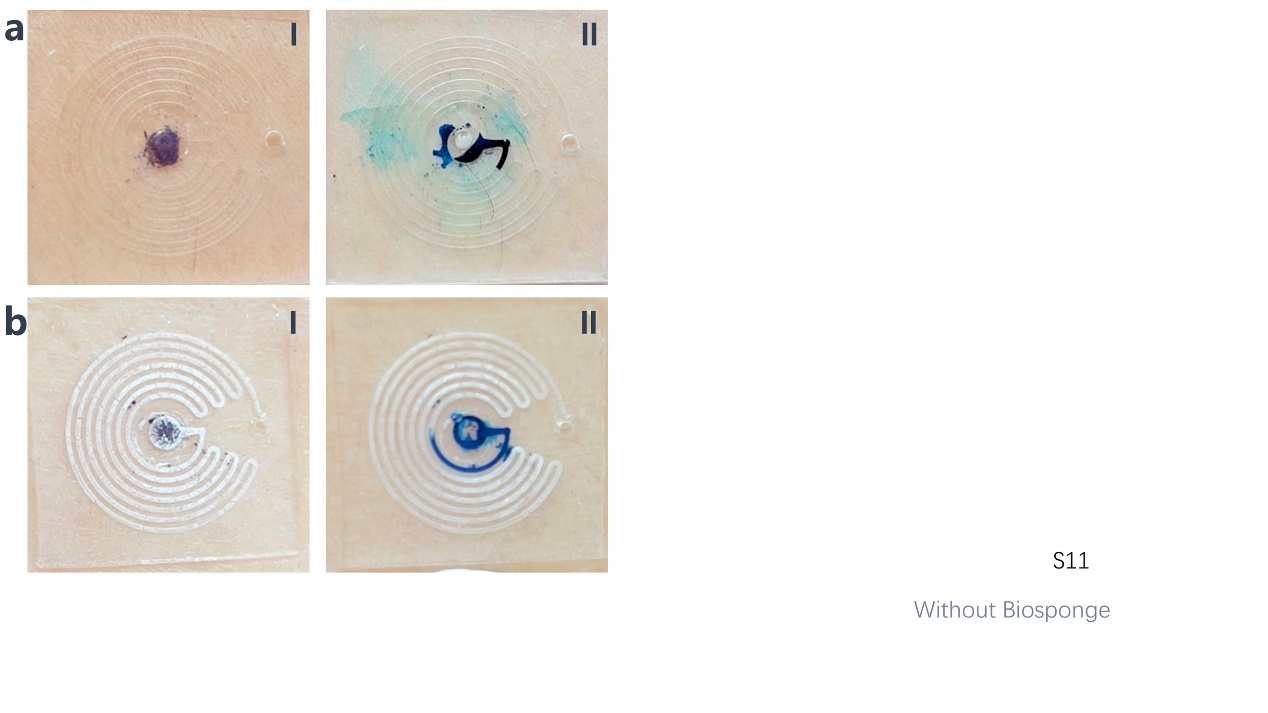


Figure. S12. A comparative analysis over an extended time interval of natural sweat harvesting efficacy.

Contrasting the conventional epidermal microfluidic patch (Group a) with the biosponge-equipped counterpart (Group b) in the context of routine sweat harvesting reveals distinct performance disparities. In Group A (a), lateral leakage around the inlet well is evident, attributed to insufficient driving pressure and hydraulic pressure loss. Contrastingly, within Group B (b), the enhanced patch exhibits adept sample entrapment capabilities, with no observed leakage or sample migration.


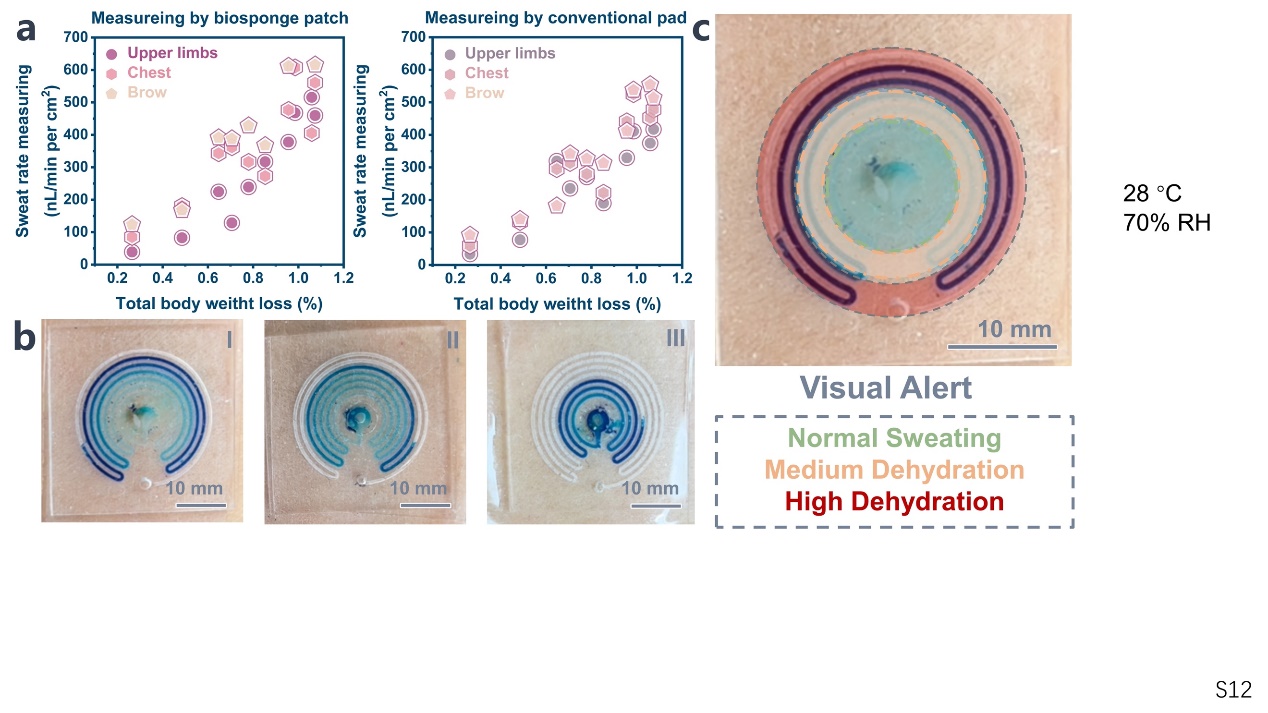


Figure. S13. Monitoring perspiration for visual alert regarding dehydration or rehydration status.

(a) A correlation manifests between the sweat rate per monitored area, as determined through biosponge-equipped patches, and the corresponding values obtained by conventional pads across three distinct sweat rate regions. (b) Three representative sweat collection volumes, indicative of the subject's dehydration patterns, are designated as follows: brow (I), chest (II), and upper limbs (III). These specific regions cater to distinct secretion rates, delineated as fast, medium, and slow. (c) The quantified analysis of captured visual regions represents a user-friendly tool, a "wireless" method for promptly gauging dehydration risks or maintaining optimal electrolyte balance during events.


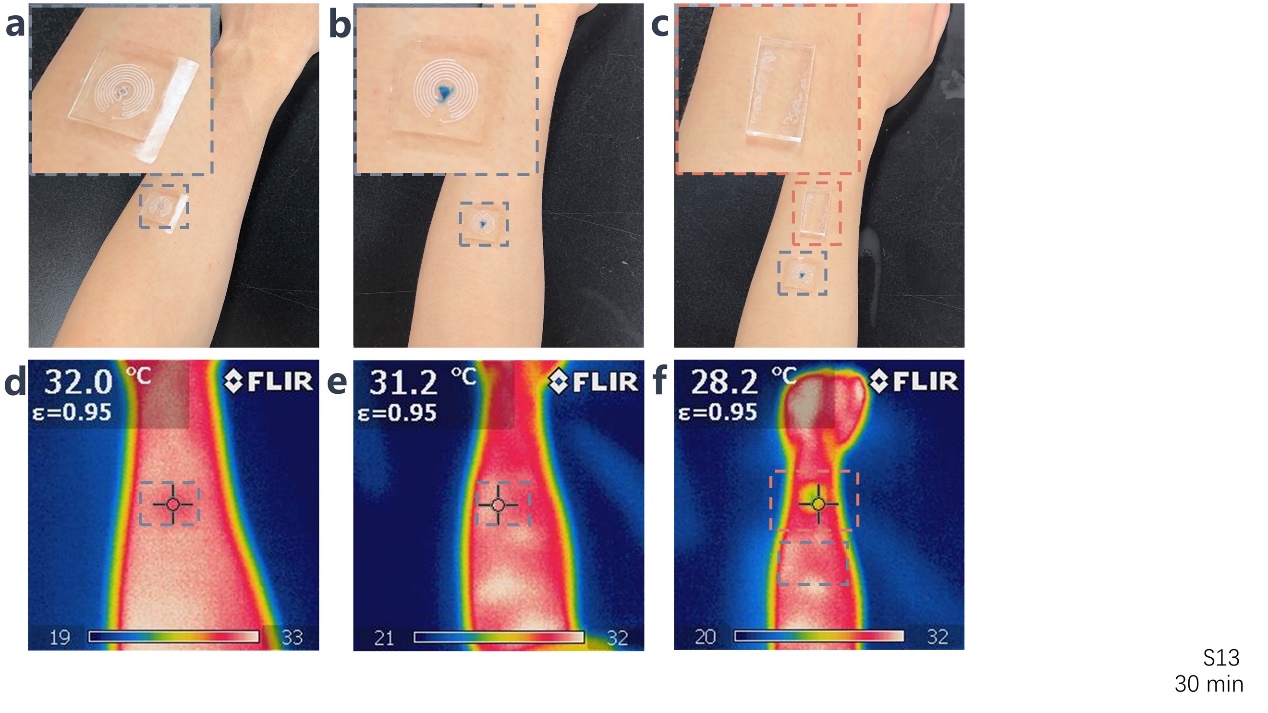


Figure. S14. Heat generation analysis.

The thermal analysis of heat generation during on-body patch attachment is exemplified through thermal infrared images captured at 10 minutes (a) and 60 minutes (b) into the application of a thin patch. Remarkably, these images reveal a marginal variance in local skin surface temperature induced by the patch. In contrast, (c) indicates that a thick patch may impede heat generation or thermal diffusion, introducing a noteworthy consideration for its potential impact on sweating rates.


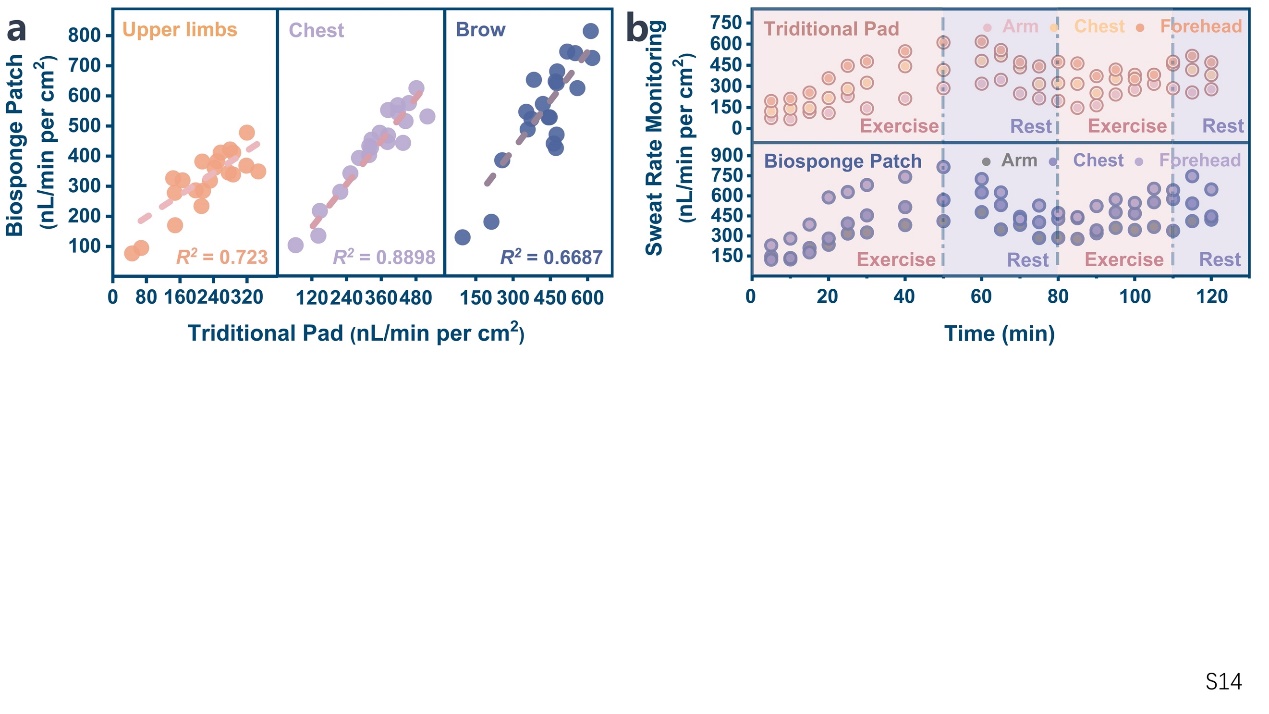


Figure. S15. Sweat monitored comparison of the direct visual image computation and conventional gravimetric approach.

(a) The interrelation between delineated sectors, as observed through the biosponge-equipped patch, and the conventional pad. The biosponge patch yielded a favorable correlation across three distinct perspiration rates, registering an approximately 1.5-fold amplification compared to measurements derived from the conventional method. (b) The monitoring of cumulative local sweat rates over time, gauged from selected regions using either a microfluidic device or a conventional pad, transpired throughout intervals of moderate exercise, rest, and a subsequent repeat event. (The upper limbs denote a gradual perspiration rate, the chest signifies a moderate rate, and the brow denotes a rapid perspiration rate.)


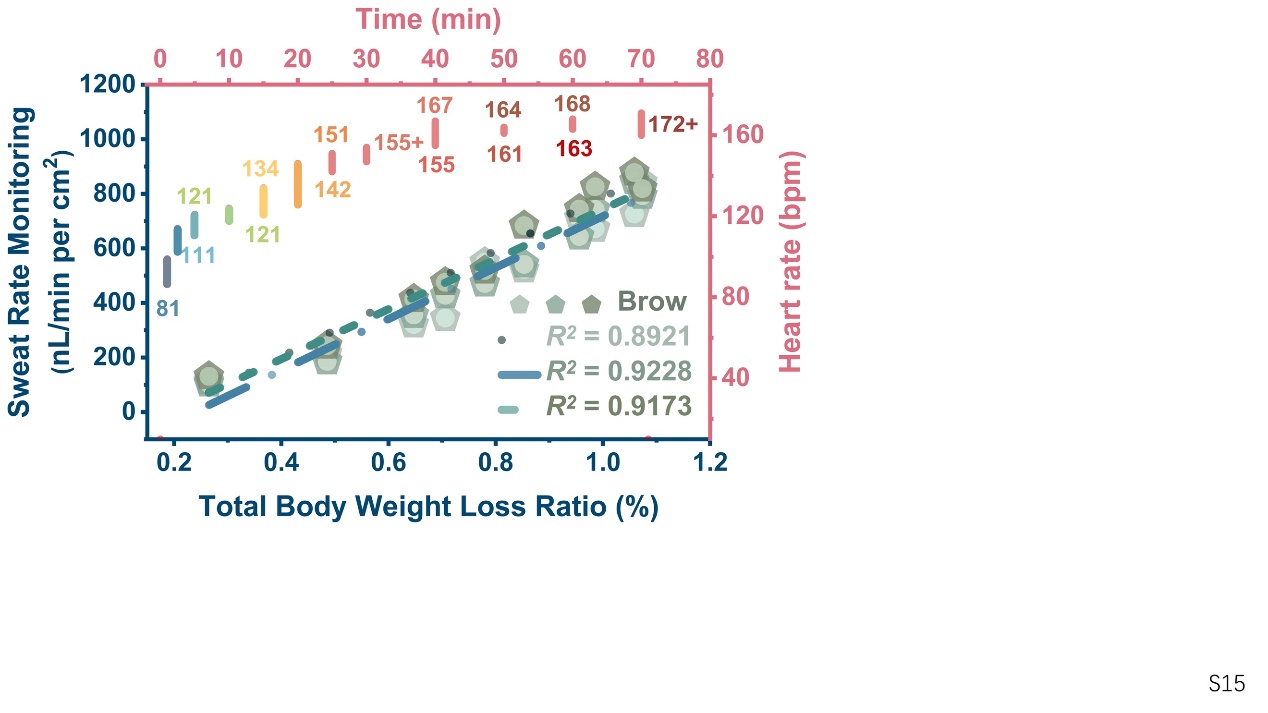


Figure. S16. The correlation between in situ sweat perspiration rate versus total body loss ratio monitored by biosponge patch.

During moderate exercise, an elevated sweat rate, coupled with an increased heart rate, exhibited a substantial correlation with the overall percentage of body weight loss, indicative of potential dehydration events.


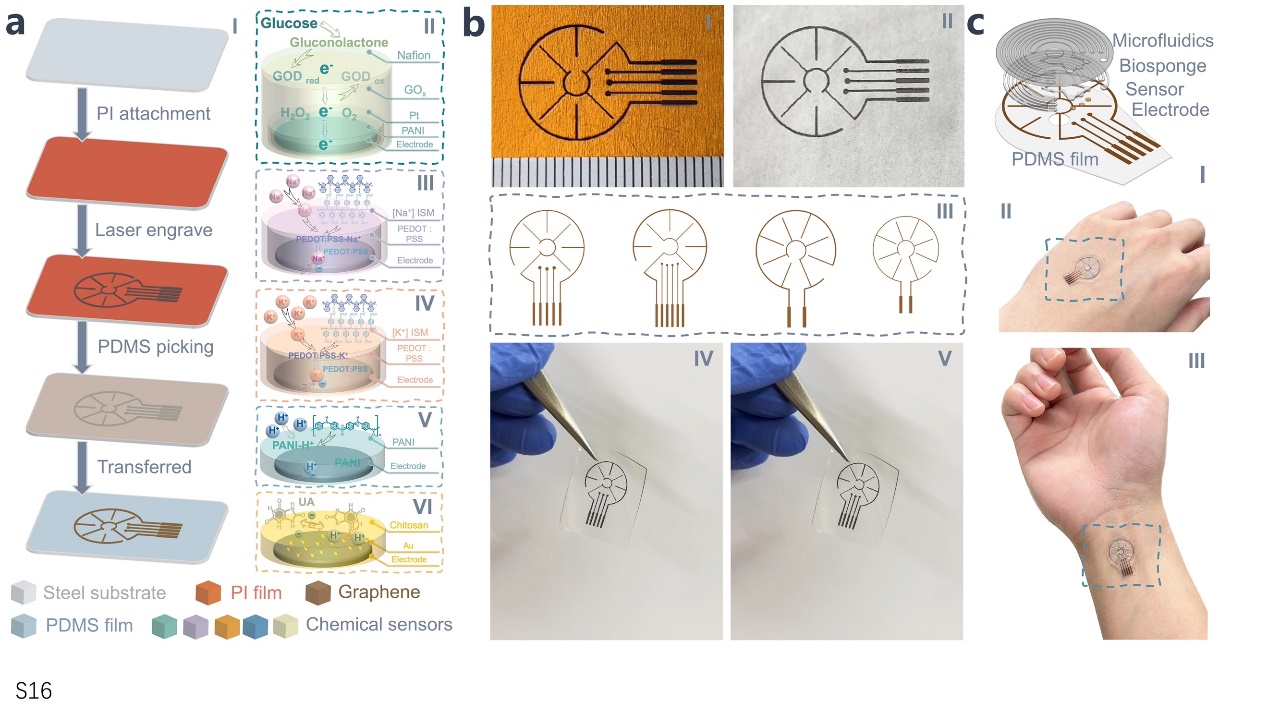


Figure. S17. A schematic depiction delineates the fabrication process of a flexible substrate featuring customized circuits and functionalized biosensors.

(a) The transprint method involves laser-engraving electrode circuits onto a flexible substrate, leading to the fabrication of biosensors designed for glucose, ion-selective membrane (ISM) measurements of [Na^+^] and [K^+^], pH sensing, and uric acid detection. (b) Optical images showcase the transprinted electrode component on Polymide substrate (I) and PDMS substrate (II), as well as custom-designed electrode variants (III). A washing test of the electrode attached to the PDMS substrate is depicted, showing the pristine state (IV) and post-water-washing (V). (c) An enlarged detailed view displays the assembly of the biosponge-equipped microfluidic patch (I), accompanied by optical images of its placement on the subject's wrist (II) and the back of the hand (III).


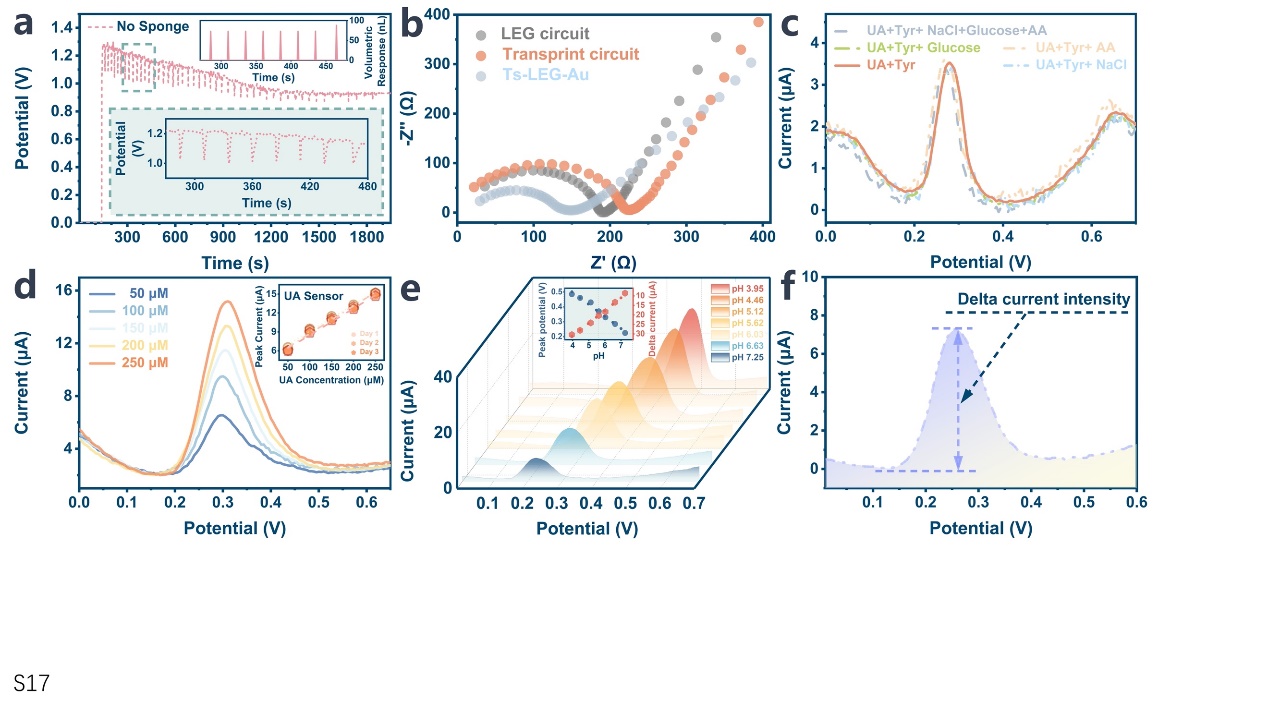


Figure. S18. Sensing performance evaluation of sweat rate sensor and uric acid sensor.

(a) The sweat rate sensor demonstrates its functionality through a plotted graph depicting the incremental volume filled inside the microchannel over time. This plot is generated under the condition of a constant flow rate of 10 mM NaCl solutions at 200 nL/min. The incremental volume specifically denotes the additional fluid filled between two adjacent radial electrodes. (b) An electrochemical impedance comparison of the as-fabricated electrode. In contrast to the pristine laser-engraved counterpart, the transprinted electrode exhibits negligible impedance change. (c) The uric acid sensor exhibits selectivity against common interferents in sweat, remaining minimally affected by the interference posed by other biomarkers present in sweat. (d) The electrocatalytic activity and reproducibility of the uric acid sensor in the direct oxidation of uric acid at physiological concentrations are evaluated. Insets accompanying the assessment include routinely tested calibration plots conducted daily. (e) The sensor response exhibits a correlation with the shift of uric acid oxidation peaks in solutions with varying pH levels. The inset features linear fittings of pH values against uric acid oxidation peak potentials and peak current densities, forming the basis for modeling the relationship between pH values and UA concentration. (f) a single Differential Pulse Voltammetry (DPV) curve extracted from (e) uric acid sensor response serves as a model illustration, showcasing the simultaneous determination of both pH value and UA concentration.


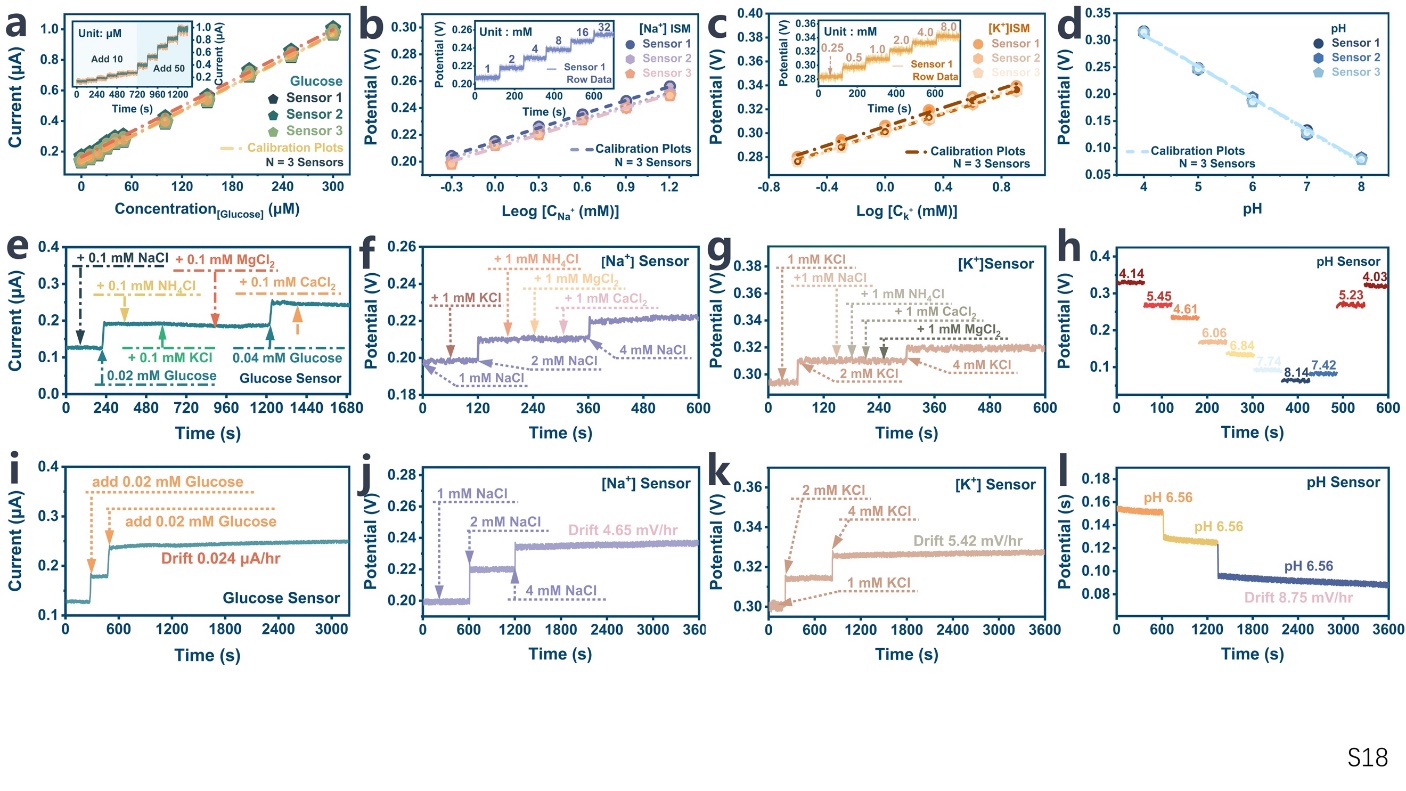


Figure. S19. Additional sensing performance of chronoamperometric sensor and potentiometric sensor.

Reproducibility of the sensing performance in response to (a) glucose (b) sodium, (c) potassium, and (d) pH levels. A sensor selectivity study investigates the sensing performance in response to (e) glucose, (f) sodium, and (g) potassium. Step voltage responses to (h) pH value alternations are examined. Long-term stability and drift analysis of the sensing performance in response to (i) glucose (j) sodium, (k) potassium, and (l) pH levels. Test results and linearity of the electrochemical sensors are established with standard sample solutions, employing an electrochemical workstation at 25 °C.


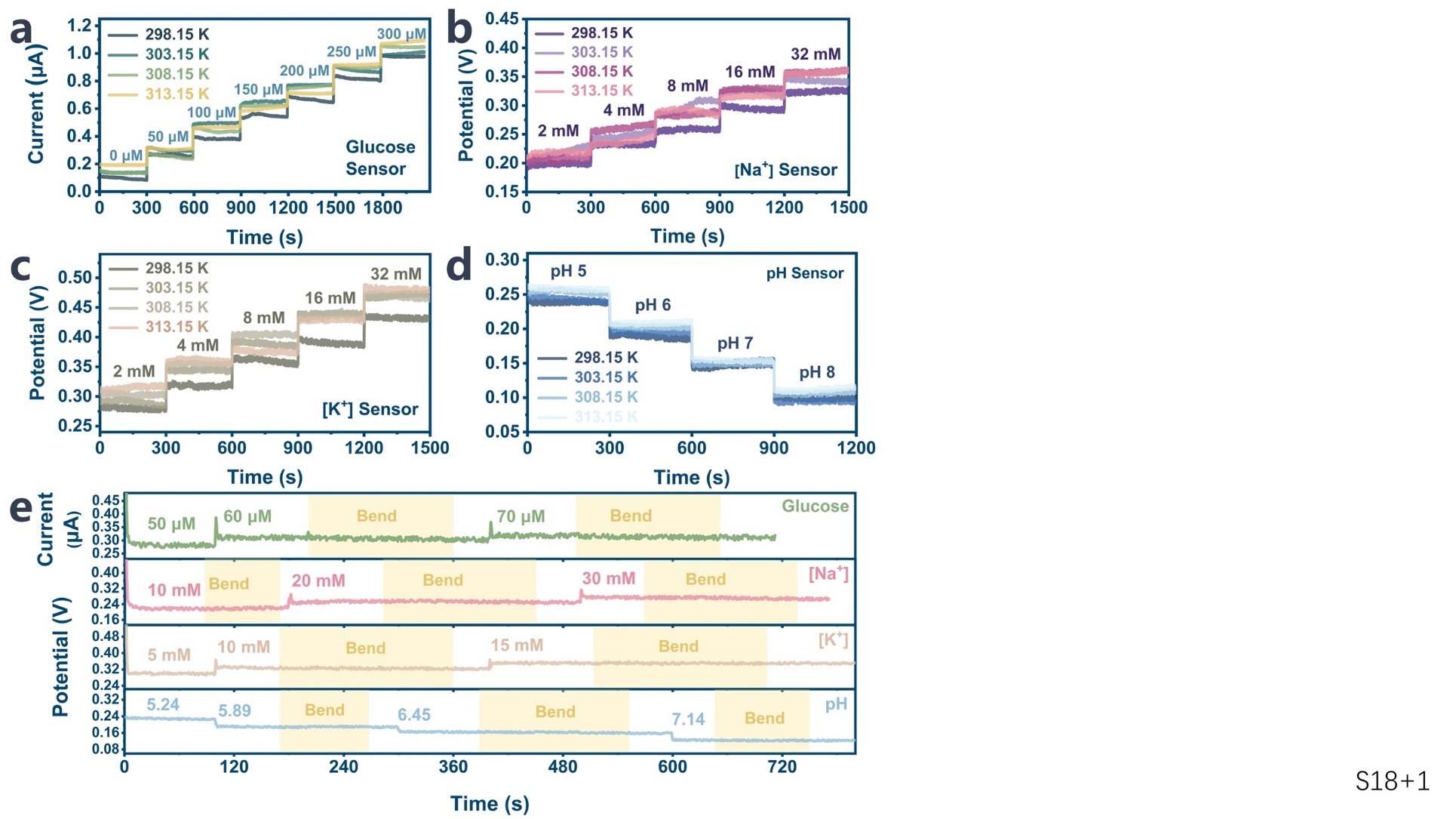


Figure. S20. Additional sensing performance of chronoamperometric sensor and potentiometric sensor.

Temperature dependence of sensor response of (a) glucose (b) sodium, (c) potassium, and (d) pH levels. (e) The patch adhered to an elastic simulated skin for long-term stability and bending motion drift analysis to mimic real-life scenarios.


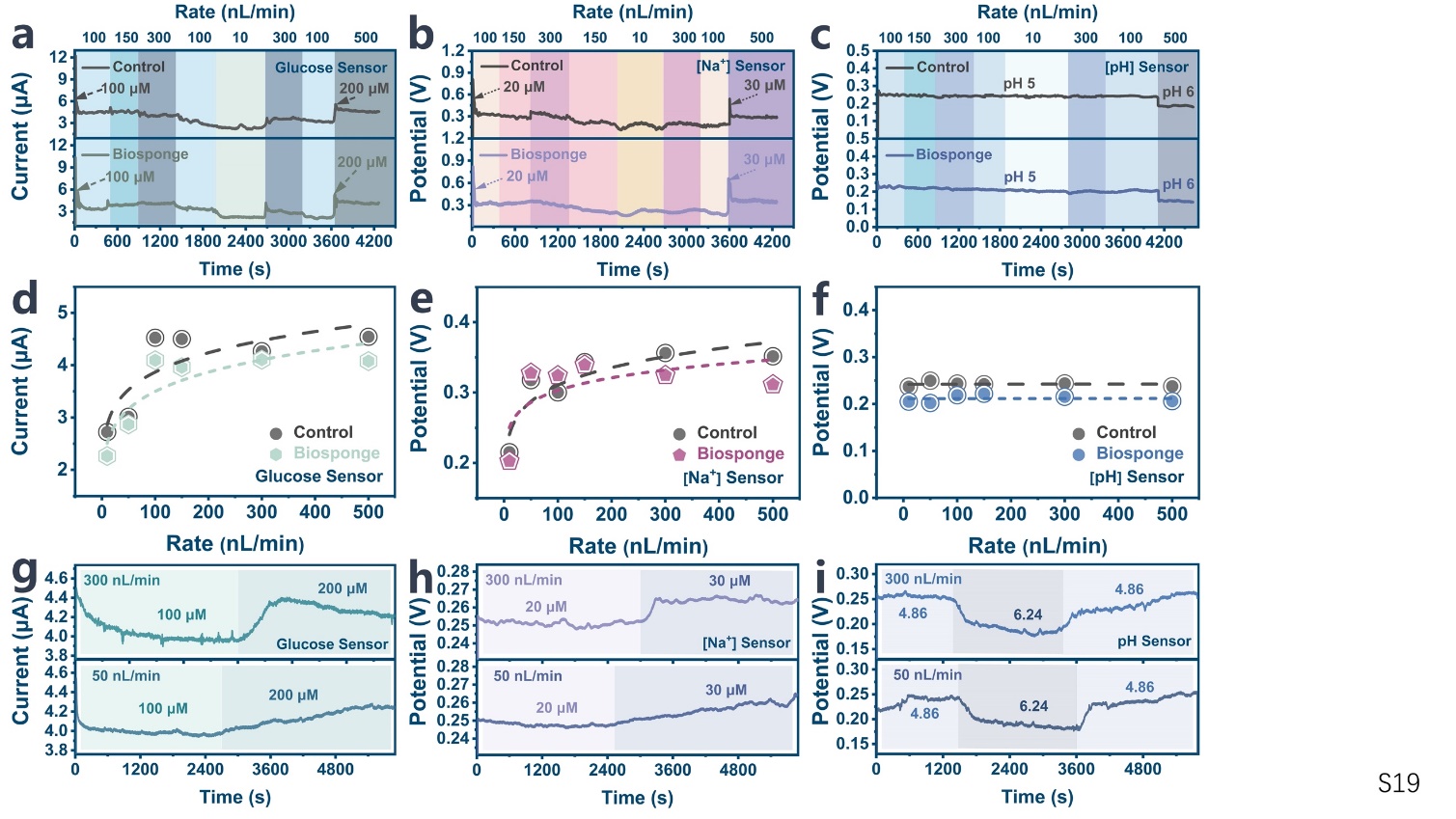


Figure. S21. Investigation of flow rate dependence of integrated biosensors within the microfluidic channel and biosponge microchannel.

Mass transfer limitations impart a discernible impact on the surface of (a) glucose, (b) sodium, and (c) pH sensing sensors with varying flow rates. Calibration plots for the (d) glucose, (e) sodium, and (f) pH sensing sensors, established at different flow rates pre-on-body testing, serve as the baseline for subsequent evaluations. Biosponge microchannel influence measurement on (g) glucose (h) sodium, and (i) pH sensing sensors inside the microfluidic channel, encompassing prolonged periods of constant slow and fast flow rates.


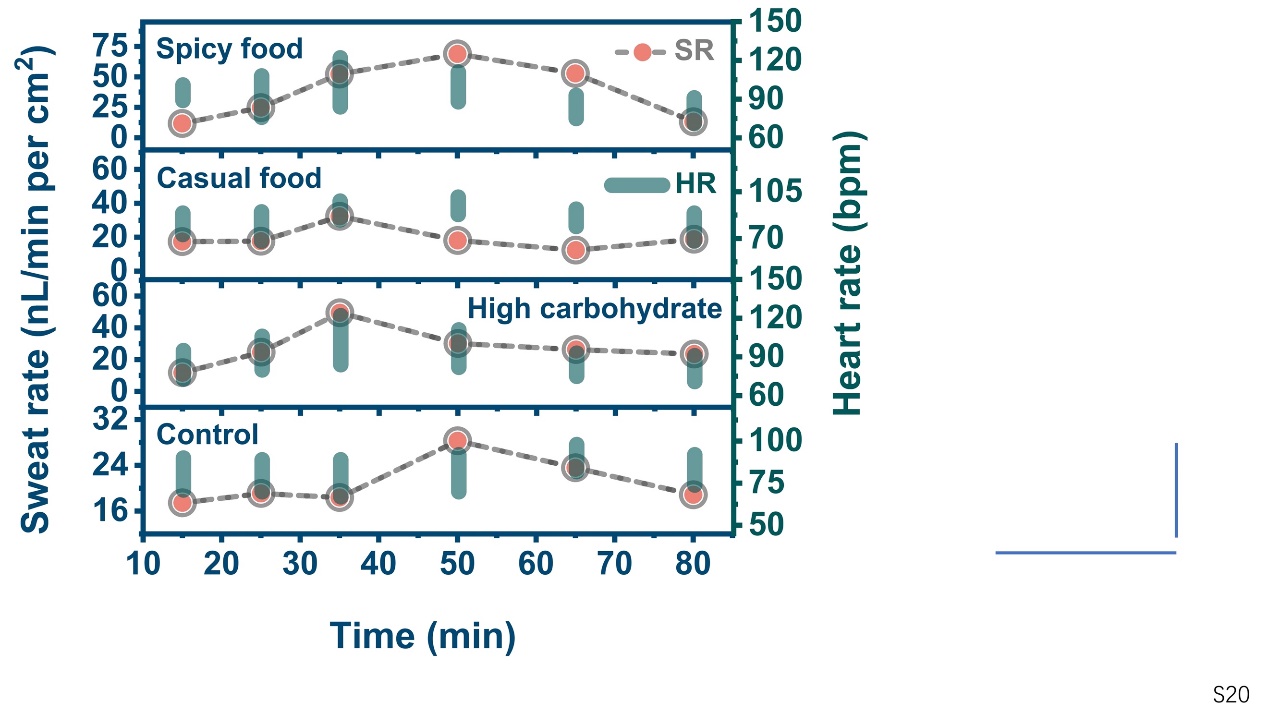


Figure. S22. In situ sweat assessment is employed to discern stress-induced events.

Elucidating stimuli such as diverse food intake on sweat behavior and heart rate.


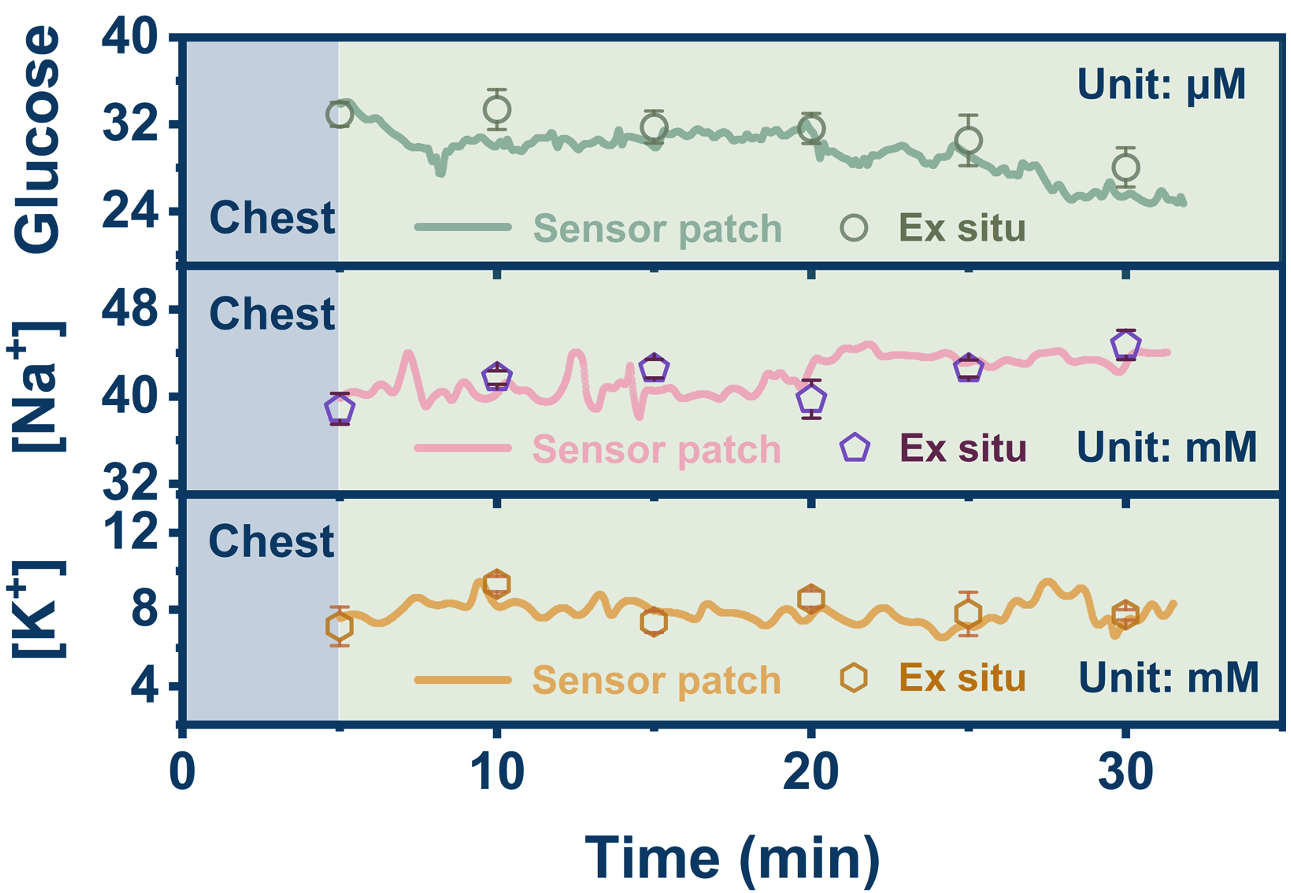


Figure. S23. On-body health monitoring with two detection systems, biosponge microfluidic patch versus standard laboratory detection.

Monitoring results encompass variations in target electrolytes ([Na^+^] and [K^+^] ions) and glucose levels in sweat over time, in all graphs, hollow dots represent sweat measurements obtained ex-situ using a commercial or laboratory sensor. Sweat was collected by centrifuging at 2,500×g for 2 minutes from absorbing pads. The monitoring process was divided into two phases: Warm-up secretion preparation (Blue section) and initiation of sweat biomarkers monitoring (Green section). (*n* = 6, error bars represent the SD)

Table S1.

Absolute regional median perspiration rates under moderate exercise, natural rates, and biosponge patch-monitored rates facilitate practical measurement of sweat rates over different body sites during various time scales. The **Monitored readouts** are calculated by considering multiple measurements of instantaneous sweat rate at each tested body location.

|  | **Body region** | **Median sweat rate (sensitive) ^[37]^** | **Monitored sweat rate (insensitive)** | **Inlet well diameter** | **Natural sweat rate ^[6]^** |
| --- | --- | --- | --- | --- | --- |
|  |  | **nL/min per cm^2^** | **nL/min per cm^2^** | **mm** | **nL/min per cm^2^** |
| **Upper** | Forehead | 680-1360 | 220-400 | 3 | 20-500 |
|  | Neck | 340-510 | 90-140 | 3 | 10-150 |
|  | Shoulder | 340-425 | 10-40 | 3 | 10-40 |
|  | Arm | 340-425 | 30-110 | 3 | 10-150 |
| **Down** | Thigh | 340-510 | 25-70 | 3 | 10-40 |
|  | Calf | 340-680 | 20-60 | 3 | 10-40 |
|  | Feet | 170-510 | 20-100 | 3 | 10-100 |
| **Rigid** | finger | 85-255 | 65-200 | 2 | 20-200 |

Movie S1.

COMSOL Multiphysics simulation: dynamic liquid advancement in a superhydrophilic biosponge microchannel facilitated by capillary action and surface wetting.

Movie S2.

Stretching cycling showcase of the serpentine-shaped biosponge-embedded microchannel.

Movie S3.

Demonstration of superior liquid sampling efficiency in a biosponge-embedded microfluidic system at the same injection speed.

References and notes

[1] K. Sato, F. Sato, *Am. J. Physiol.-Regul. Integr. Comp. Physiol.* **1983**, *245*, R203.

[2] I. J. Schulz, *J. Clin. Invest.* **1969**, *48*, 1470.

[3] K. Wilke, A. Martin, L. Terstegen, S. S. Biel, *Int. J. Cosmet. Sci.* **2007**, *29*, 169.

[4] M. J. Buono, N. V. L. Lee, P. W. Miller, *J. Physiol. Sci.* **2010**, *60*, 103.

[5] J. T. Reeder, J. Choi, Y. Xue, P. Gutruf, J. Hanson, M. Liu, T. Ray, A. J. Bandodkar, R. Avila, W. Xia, S. Krishnan, S. Xu, K. Barnes, M. Pahnke, R. Ghaffari, Y. Huang, J. A. Rogers, *Sci. Adv.* **2019**, *5*, eaau6356.

[6] N. A. Taylor, C. A. Machado-Moreira, *Extreme Physiol. Med.* **2013**, *2*, 4.
